# Performance evaluation of handheld Raman spectroscopy for cocaine detection in forensic case samples

**DOI:** 10.1002/dta.2993

**Published:** 2021-01-07

**Authors:** Ruben F. Kranenburg, Joshka Verduin, Renee de Ridder, Yannick Weesepoel, Martin Alewijn, Marcel Heerschop, Peter H.J. Keizers, Annette van Esch, Arian C. van Asten

**Affiliations:** ^1^ Forensic Laboratory Dutch National Police, Unit Amsterdam Amsterdam The Netherlands; ^2^ Van't Hoff Institute for Molecular Sciences University of Amsterdam Amsterdam The Netherlands; ^3^ Wageningen Food Safety Research Wageningen University and Research Wageningen The Netherlands; ^4^ Dutch Customs Laboratory Amsterdam The Netherlands; ^5^ National Institute of Public Health and the Environment (RIVM) Bilthoven The Netherlands; ^6^ Netherlands Forensic Institute (NFI) The Hague The Netherlands; ^7^ Co van Ledden Hulsebosch Center (CLHC), Amsterdam Center for Forensic Science and Medicine Amsterdam The Netherlands

**Keywords:** illicit drug analysis, on‐scene detection, portable device, Raman spectrometer, TruNarc

## Abstract

Handheld Raman spectroscopy is an emerging technique for rapid on‐site detection of drugs of abuse. Most devices are developed for on‐scene operation with a user interface that only shows whether cocaine has been detected. Extensive validation studies are unavailable, and so are typically the insight in raw spectral data and the identification criteria. This work evaluates the performance of a commercial handheld Raman spectrometer for cocaine detection based on (i) its performance on 0–100 wt% binary cocaine mixtures, (ii) retrospective comparison of 3,168 case samples from 2015 to 2020 analyzed by both gas chromatography–mass spectrometry (GC–MS) and Raman, (iii) assessment of spectral selectivity, and (iv) comparison of the instrument's on‐screen results with combined partial least square regression (PLS‐R) and discriminant analysis (PLS‐DA) models. The limit of detection was dependent on sample composition and varied between 10 wt% and 40 wt% cocaine. Because the average cocaine content in street samples is well above this limit, a 97.5% true positive rate was observed in case samples. No cocaine false positives were reported, although 12.5% of the negative samples were initially reported as inconclusive by the built‐in software. The spectral assessment showed high selectivity for Raman peaks at 1,712 (cocaine base) and 1,716 cm^−1^ (cocaine HCl). Combined PLS‐R and PLS‐DA models using these features confirmed and further improved instrument performance. This study scientifically assessed the performance of a commercial Raman spectrometer, providing useful insight on its applicability for both presumptive detection and legally valid evidence of cocaine presence for law enforcement.

## Graphical abstract

The performance of a handled Raman spectrometer for cocaine detection is evaluated by validation samples, spectral assessment, and retrospective comparison. Raman and GC–MS results of 3,168 cases samples from 2015 to 2020 were compared. Limits of detection were dependent of sample composition and varied between 10 and 40 wt% cocaine. A 97.5% true positive rate was observed in case samples. Instrument performance was assessed and further improved by a combined partial least square regression (PLS‐R) and discriminant analysis (PLS‐DA) model.

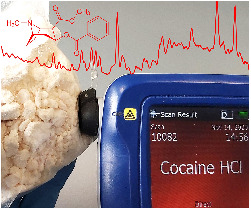

## 1 INTRODUCTION

Within the illicit drug market, cocaine is one of the most prevalent substances with a global estimated annual production of around 2,000 metric tons.^1^ Consequently, cocaine seizures and subsequent judicial actions are a daily routine for many investigative authorities throughout the world. Rapid and reliable on‐scene detection methods are required to confirm the first suspicion and determine whether substances should be seized, or suspects should be taken into custody. Many current standard methodologies for cocaine detection comprise of colorimetric spot tests based on the formation of a blue colored cobalt(II) thiocyanate complex in presence of cocaine.^2^, ^3^ Despite being fast and inexpensive, colorimetric tests have several limitations. First, such tests are destructive and thus require opening the packaging of the (yet) unknown substance. Without adequate precautions, this can impose a health risk when highly potent substances such as fentanyl derivates are encountered. Second, colorimetric tests are known to yield false positive (FP) results on some common pharmaceuticals such as lidocaine, levamisole, and promethazine.^4^, ^5^ Third, individual colorimetric tests are only available for a limited set of common drugs meaning that they are only selective towards a limited number of substances. This may lead to false negative (FN) results when the incorrect test is performed or a controlled substance is encountered for which no test is available.^3^

Other indicative testing strategies for on‐site cocaine detection using handheld devices include electrochemical analysis,^6^ (near) infrared spectroscopy,^7^, ^8^, ^9^, ^10^ and Raman spectroscopy^11^, ^12^, ^13^, ^14^ where the latter two offer possibilities to analyze substances directly through packaging material (when this material is sufficiently transparent). Over the last decade, Raman spectroscopy has become a viable tool in forensic chemistry, and its applications have been reviewed multiple times.^9^, ^13^, ^14^, ^15^ In addition to laboratory‐grade instrumentation, various commercial handheld spectrometers with a specific focus on the operation by first responders, law enforcement officers and crime scene investigators have come to market. The TruNarc handheld Raman spectrometer was introduced in 2012 as the first commercial instrument designed specifically for forensic drug detection. Most handheld instruments are tailored for on‐scene use by untrained personnel using single‐button operation and subsequent readback of the (often binary) result on a display. Although efficient and easy to implement, a drawback of this approach is the lack of demonstrable evidential value and validation. Built‐in data processing, library search and matching procedures and identification criteria are often proprietary and not fully disclosed by the manufacturer. As a result, the instrument operates as a “black box.” Besides, manufacturers generally may not have access to a vast amount of representative case samples for validation, and therefore only limited studies are reported on the performance of the built‐in functionality of these devices.^16^, ^17^, ^18^ In situations in which the result is only used as an indication, this can be acceptable. However, when judicial actions such as seizure or custody are undertaken, this is unwanted as a FP outcome can have severe adverse consequences on the suspects involved, whereas a FN outcome can lead to serious crime remaining undetected. Additional insight into the performance and selectivity of such handheld devices is also crucial for optimal detection strategies in forensic laboratories. When multiple (orthogonal) handheld techniques are available, these can provide complementary evidence and ultimately might eliminate the need for confirmatory analysis within the laboratory. In this way, tremendous process efficiency could be achieved in the judicial chain as both time and money are saved by the elimination of transport, administration, laboratory analysis and reporting steps. To that end, the overall turnover time from seizure to conviction could be decreased dramatically.

According to international guidelines provided by the Scientific Working Group on Drugs (SWGDRUG), Raman spectroscopy is considered a Category A technique providing the highest level of selectivity through structural information.^19^ In this way, an analytical scheme based on Raman spectroscopy and a colorimetric test may suffice for unambiguous identification suitable as court evidence. However, dedicated requirements are described for handheld Raman spectrometers such as the instrument used in this study. The handheld has “to be assessed and validated for this purpose to ensure that the resolution and spectral range provide sufficient structural information to achieve the selectivity requirement of a Category A technique.”^20^ Additionally, spectral data need to be reviewable. Currently, in The Netherlands, handheld Raman spectrometers are only used for presumptive testing, and subsequent laboratory testing (e.g., gas chromatography–mass spectrometry [GC–MS] analysis) is required for court evidence. In certain circumstances, however, local policies (e.g., at dance festivals) can lead to a financial settlement if a suspect confesses the possession of a user quantity of illicit drugs after only a presumptive test by a handheld Raman spectrometer. In this way, no further laboratory testing is performed, and the Raman spectrometer is indirectly used for absolute identification purposes.

Nevertheless, one major limitation of Raman spectroscopy in forensic drug detection is its sensitivity. Mixtures—which cocaine‐containing case samples often are—can be challenging as the analyzed signal contains features from all substances present, which may complicate database searches. Also, the presence of fluorescent dyes or impurities in a sample, even at low concentration, can obscure Raman signals and prevent identification of the compound of interest. A possible strategy to reduce the influence of fluorescence is the use of higher wavelength excitation lasers; however, this also comes at the cost of a reduced Raman signal.^21^ A 785‐nm excitation laser was reported as a good compromise between sensitivity and background fluorescence.^9^ Surface‐enhanced Raman spectroscopy (SERS) is a technique that could significantly increase sensitivity, although the use of dedicated consumables is required and SERS spectra are different from native Raman spectra complicating its applicability in on‐scene use.^9^, ^22^, ^23^ To optimally exploit the spectral features in the Raman signal, chemometric approaches can be applied. Ali and Edwards demonstrated the use of principal component analysis (PCA) on Raman spectra to distinguish clothing samples impregnated with either cocaine, MDMA or amphetamine.^11^ Both Muhamadali et al.^23^ and Omar et al.^24^ applied PCA for group classification of various new psychoactive substances (NPS). The spectral selectivity of Raman spectroscopy for pure drug substance detection and classification was demonstrated by Calvo Castro et al., who analyzed a large set of 478 NPS and visualized several group‐specific features using PCA.^25^ Supervised chemometric approaches were applied for classification or quantification issues within and outside the forensic field. Jiménez Carvalo et al.^26^ applied partial least squares (PLS) discriminant analysis (PLS‐DA) and regression (PLS‐R) on Raman spectra for olive oil classification and quantification. Lê et al.^27^ applied PLS‐R for gemcitabine quantification in plastic bags containing solutions of this intravenous drug. Ellis et al. quantified the methanol content in spirits using PLS‐R.^28^ All these studies were performed by directly analyzing the samples though various packaging materials (i.e., plastic bags and glass vials). Within the forensic field, Katainen et al. demonstrated the quantitative performance of Raman spectroscopy for amphetamine case samples using a PLS‐R model.^29^ Regarding cocaine, De Oliveira Penido and coworkers quantified cocaine base (crack cocaine) mixtures with sodium carbonate and either caffeine or lidocaine from 10 to 100 wt%.^30^ Bedward et al. successfully quantified cocaine HCl concealed in food matrices such as cake mix in concentrations above 20 wt% using PLS‐R.^31^ It must be noted that all these studies regarding cocaine detection were performed on benchtop laboratory instruments. However, for on‐scene indicative detection of street samples, these benchtop instruments are not practical, and cheaper and portable spectrometers are needed.

To our knowledge, no earlier work has reported the use of chemometric‐based cocaine detection using a handheld Raman spectrometer. In addition, this is the first time the performance, and overall applicability of a handheld Raman spectrometer is assessed on a large set of actual case samples. In this work, we evaluated the performance of a commercial 785‐nm handheld Raman narcotics analyzer on 0–100 wt% mixtures of cocaine HCl. Results are presented for cocaine mixed with eight commonly encountered cutting agents, each showing a different limit of detection (LOD) for cocaine due to differences in spectral features. Performance of the built‐in “black box” software was further assessed using both a PLS‐DA and a PLS‐R model to detect cocaine by comparing the predicted cocaine concentrations against a threshold level to optimize FP and FN rates. We furthermore provide a retrospective comparison of 3,168 Raman results from case material (analyzed between 2015 and 2020) and their corresponding GC–MS results.

## 2 MATERIALS AND METHODS

### 2.1 Materials

Levamisole HCl (>99 wt%), paracetamol (acetaminophen, pure), and *myo*‐inositol (>99 wt%) were obtained from Sigma Aldrich (St. Louis, MO, USA). Caffeine HCl (pure) was obtained from AppliChem (Darmstadt, Germany), phenacetin (research grade) was purchased from Serva (Heidelberg, Germany), and procaine HCl (pure) was acquired from Merck (Darmstadt, Germany). Food grade lactose and mannitol were obtained from a local smart shop. Cocaine HCl originated from a >98 wt% purity case sample provided by the Amsterdam Police Laboratory.

Ninety cocaine case samples were provided by the Netherlands Forensic Institute (NFI) and originated from seizures by the Dutch National Police in 2017 (Table 1). Forty non‐cocaine‐containing samples were provided by the Amsterdam Police Laboratory. The general characteristics of these sets are as follows: 58 cocaine HCl samples (average cocaine content 64.4 wt%, range 85.5–19.1 wt%); 32 cocaine base samples (average cocaine content 75.4 wt%, range 99.4–31.5 wt%); 20 pure adulterants, cutting agents or uncontrolled pharmaceuticals; 10 mixtures of common cutting agents; 10 common controlled substances with a white powdery appearance (e.g., amphetamine, mephedrone, MDMA, and methamphetamine). Full details of these sets are reported elsewhere.^7^

**TABLE 1:** Overview of sample sets and their purpose in this study

Sample set (section)	Number of samples	Number of cocaine HCl samples (type, %wt range)	Number of negative samples	Year or year range	Purpose in study (section)
NFI case samples (2.1)	90	90 (58× HCl, 32× base, 99%–19%)	0	2017	Performance evaluation built‐in software (3.1.1) Training set for model design and evaluation (3.3.1)
Amsterdam police case samples (2.1)	53	1 (base, 100%)	52	2019–2020	Performance evaluation built‐in software (3.1.1) Training set for model design and evaluation (3.3.1)
Binary mixtures (2.2)	88	80 (HCl, 100%–10%)	8	N.A.	Performance evaluation built‐in software (3.1.1) Training set for model design and evaluation (3.3.1)
Retrospective analysis (2.5)	445	245^a^	200	2015–2020	External validation set (3.1.2 and 3.3.3)
Total	676	416	260		N.A.

Abbreviations: N.A., not applicable; NFI, Netherlands Forensic Institute.

^a^ Cocaine type and concentration unknown.

Thirteen case samples provided by the Amsterdam Police Laboratory were included in this study that were identified to contain 4‐hydroxybutyric acid (GHB), both dried and in aqueous solution; heroin; cocaine base; 2‐methylmethcathinone (2‐MMC); 3‐methylmethcathinone (3‐MMC); 3‐methylethcathinone (3‐MEC); 4‐methylethcathinone (4‐MEC); 2‐(4‐bromo‐2,5‐dimethoxyphenyl)ethanamine (2C‐B); 6‐(2‐aminopropyl)benzofuran (6‐APB); 2‐fluoromethamphetamine (2‐FMA); 3‐chloromethcathinone (3‐CMC); and 4‐chloromethcathinone (4‐CMC) (Table 1).

### 2.2 Sample preparation

Binary mixtures of cocaine HCl and the eight cutting agents from section 2.1 were prepared from 0 to 100 wt% cocaine by grinding and mixing the appropriate amounts to a total of 200 mg per sample in a mortar. This resulted in a set of 8 negative samples (being the pure cutting agents), 72 binary mixtures of cocaine HCl with cutting agents, and eight samples of pure cocaine HCl (Table 1). All samples were stored in 4‐ml clear borosilicate glass vials at room temperature in the dark.

### 2.3 Portable Raman data acquisition

Raman measurements were performed using two TruNarc Handheld Narcotic Analyzers from Thermo Fisher Scientific (Waltham, MA, USA). These devices operate with a 785‐nm laser at 250‐mW output power for excitation. Raman spectra were recorded at a fixed 300‐ to 1,800‐cm^−1^ wavelength range. The instrument's performance was checked at least daily using the manufacturer's built‐in suitability check by scanning the attached plastic lid. Samples were analyzed within their vials by scanning through the glass wall using the provided sample holder for vial scanning. For case samples described in Section 2.1, duplicate scans were recorded on a single instrument, and an additional single scan was measured on a second instrument, thus leading to a total of three scans per sample. All pure and binary‐mixed cocaine samples (Section 2.2) were analyzed in tenfold on a single instrument. For chemometric analysis, raw spectral data were exported in the .spc file format. Each spectral data file consisted of 1,153 variables indicating a spectral resolution of approximately 1.3 cm^−1^.

### 2.4 Reference analysis

GC–MS results used for comparison were obtained from sample solutions in dichloromethane analyzed in full‐scan MS mode on a single quadrupole instrument using the laboratory's validated methods described elsewhere.^32^, ^33^ Fourier transform infrared (FTIR) analyses were performed on a benchtop Spectrum Two spectrometer with ATR option from PerkinElmer (Waltham, MA) using a scan range of 400–1,400 cm^−1^.

### 2.5 Retrospective analysis

For retrospective analysis of the Raman spectra and external validation of the trained model, a database query was executed in the Laboratory Information Management System (LIMS) of the Amsterdam Police Laboratory. In this way, all case sample numbers were retrieved for which both a TruNarc Raman result and a GC–MS result were available. Corresponding raw spectral Raman and GC–MS data were traceable via unique identifiers in the LIMS. Raman spectra originating from the retrospective analysis were scanned by different technicians according to the manufacturer's instructions by a single scan using the point‐and‐shoot method or scanning through a layer of plastic packaging. The database query led to a total selection of 3,168 unique case samples seized and analyzed between 2015 and 2020 (Table 1). From this selection, 1,775 samples were identified as cocaine containing by ISO 17025 accredited GC–MS methods. The other 1,393 samples did not contain cocaine. This latter group consisted of a broad range of samples identified as other drugs of abuse (e.g., MDMA, amphetamine, and ketamine), adulterants or common pharmaceuticals (e.g., paracetamol, lidocaine, and levamisole) or were reported as negative (not containing a controlled substance) without an indication of its identity. Out of the 3,168 unique samples, 445 samples were randomly selected and set aside as external validation set for the PLS‐models: 200 spectra from cocaine‐containing samples that were detected as cocaine positive by the spectrometers' built‐in software (true positive, TP); 200 spectra from non‐cocaine samples that were not detected as cocaine (i.e., Raman result negative, inconclusive, or another compound detected) (true negative, TN); and 45 spectra from cocaine‐containing samples that were detected as negative by the handheld Raman instrument (FN). As no FPs for cocaine were encountered, there were no spectra to further assess in this way.

### 2.6 Multivariate statistics

#### 2.6.1 Data analysis performed by built‐in software

Spectral data were automatically processed, analyzed, and library searched by the built‐in software (TruNarc version 1.8.19062 with software version 2019 1.9.09919345) for which no details were disclosed other than a second derivative signal pre‐processing and a library consisting of three hierarchical sublibraries of controlled substances (according to US legislation); drug precursors (according to US legislation) and other substances. When a spectral match is found in a higher‐level library; this match is shown on the device's screen, and subsequent libraries are not searched.

#### 2.6.2 Customized data analysis

Preliminary data exploration using PCA, PLS‐DA, and PLS‐R was performed in Unscrambler 11 (Camo Analytics, Oslo, Norway). For subsequent modeling of the Raman data R, version 3.6.3 2020‐02‐29 (R Foundation for Statistical Computing, Vienna, Austria) was used in the RStudio environment (version 1.2.5033). For PLS, the package pls_2.7‐2^34^ was used. For the PLS‐R model, spectral preprocessing consisted of standard normal variate (SNV) normalization of the full spectrum, followed by a nine‐datapoint Savitzky–Golay (SG) smoothing. Subsequently, a 1,700‐ to 1,728‐cm^−1^ region of interest (ROI) window was selected, and baseline correction was performed by subtracting the value of the lowest data point from all data points. In the PLS‐DA model with the focus on one cocaine‐selective spectral peak, the 1,700‐ to 1,728‐cm^−1^ ROI was selected first, followed by SNV normalization, SG smoothing, and baseline correction.

A training set was created from all cocaine and non‐cocaine samples (Section 2.1) and all binary cocaine HCl mixtures and corresponding pure compounds (Section 2.2). This training set was used for both the PLS‐R and PLS‐DA model. Outliers were detected by performing PCA on all replicate spectra of a unique sample, determining the sum of PC1–3 for each spectrum, and marking spectra outside of the 99% quantile as outliers. This resulted in three out of 1,372 spectra labeled as outliers and these spectra were subsequently excluded from further analysis. PLS models were cross validated by creating 10 segments out of the training set. Replicate scans were all kept within the same segment. For the PLS‐R model, four components were used for subsequent predictions. These four components showed over 99% explained variance and optimal low cross‐validated root mean square error of prediction (RMSEP_cv_). The loading plots of these four components described both the spectral features of cocaine HCL (Component 1) and cocaine base (Component 3) as well as negative loadings for the spectral features attributed to procaine (Component 2) and ketamine (Component 4), being the only substances in the training set with a spectral peak partly present within the confined ROI. Higher PLS components were attributed to noise. For the PLS‐DA model, only the first two components were selected as their loading plots described the spectra of cocaine base and cocaine HCl (after preprocessing) and the third component was attributed to noise. The 445 spectra from retrospective analysis (Section 2.5) were not included in model design and were analyzed as native samples by the PLS models.

## 3 RESULTS AND DISCUSSION

### 3.1 Instrument performance evaluation

#### 3.1.1 Performance on binary mixtures and validation samples

Results of the handheld Raman analyzer (built‐in software evaluation) were compared with the results reported by the forensic laboratories based on their validated GC–MS‐ and FTIR‐based identification methods. The full results are shown in Tables S1–S3. For 253 out of 261 scans (97%) of the cocaine case samples, the correct cocaine type (HCl or base) was assigned. For two scans, cocaine was detected, but with the incorrect classification and for 1 scan cocaine was correctly detected, but no class was obtained for the best matching library spectrum. This latter indicates that multiple spectra of a similar reference compound might be present in the built‐in spectral library. For four replicate scans of the same sample, inconclusive results were obtained, and for one scan, a FN result was returned by the handheld software. The erroneous results originated from case samples containing a cocaine content equal to or lower than 31 wt%. These results indicate that the handheld spectrometer performs well for average cocaine samples, but limitations may arise for samples with reduced cocaine levels as encountered for instance in smuggling scenarios. The average cocaine content in seized materials in Europe often exceeds 40 wt% as reported by various drug market trend reports^35^, ^36^ implying that such a handheld analyzer could successfully be implemented for most cocaine samples. However, from a forensic point of view, it is important to focus not only on the samples that have a high probability to be encountered in casework but also objectively assess the performance at lower concentrations to determine the LOD. For the 52 non‐cocaine samples mentioned in Table 1 (other drugs, cutting agents, mixtures), most compounds were correctly detected (Table S2) except for MDMA powder, heroin, 6‐APB, and 3‐MEC. These first three substances are known to exhibit fluorescence compromising its detectability by conventional Raman spectroscopy. It must be noted that the manufacturer supplied dedicated consumables for SERS to aid detectability of MDMA and heroin. However, these consumables were not used as the aim of this study is to assess the performance of the handheld device for nondestructive and “direct scanning” purposes. The NPS substances 3‐MEC and 6‐APB produced inconclusive results that could be attributed to lacking spectra in the reference library, a known issue for novel drugs and an illustration of the need for the continuous updating of spectral libraries.

**FIGURE 1:**
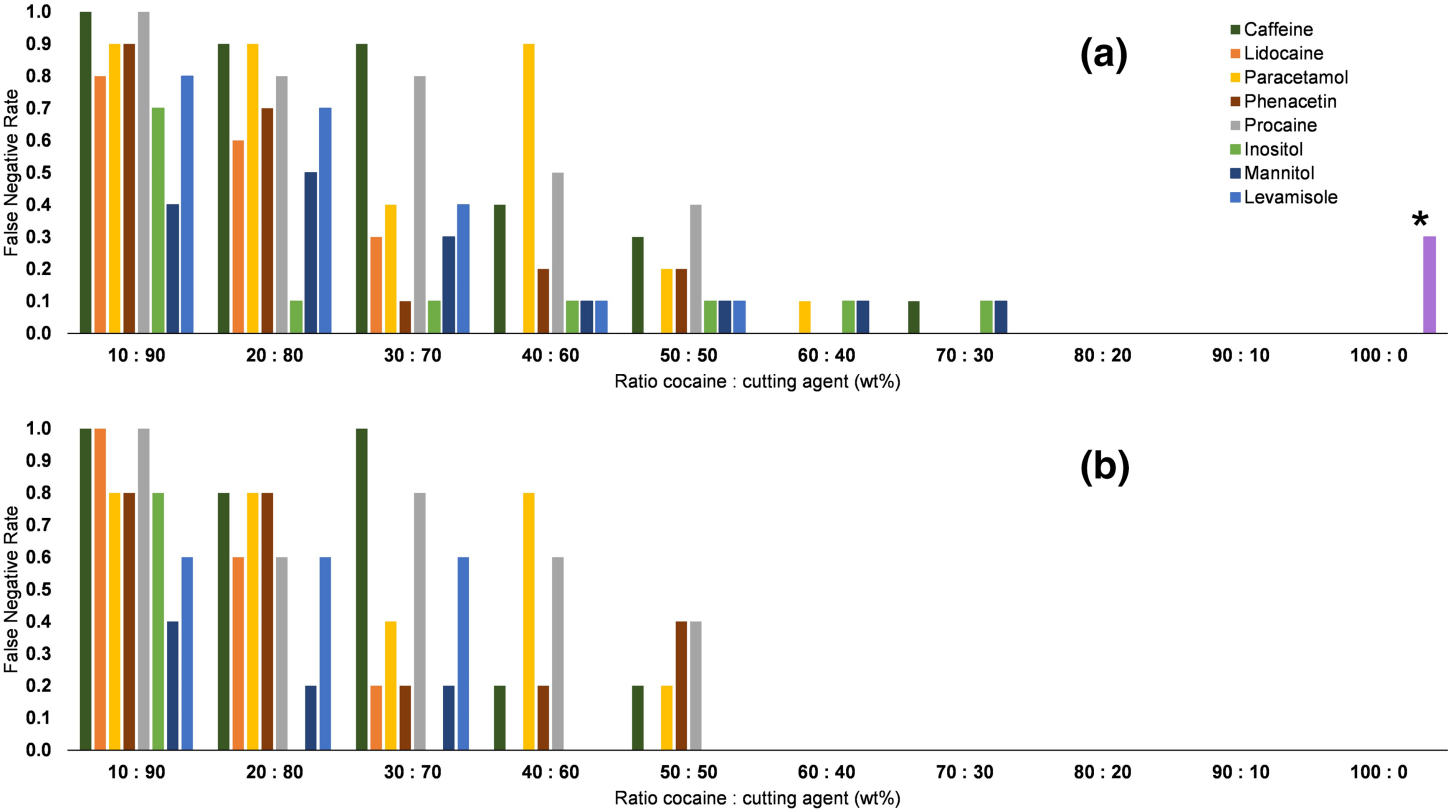
False negative rates of the TruNarc handheld Raman spectrometer for binary cocaine mixtures with eight common cutting agents at concentrations ranging from 10 to 100 wt% cocaine. Plot (a) shows results from tenfold replicates of samples scanned without special instructions and including inconclusive scans. (*) indicates inconclusive results for pure cocaine (three out of 80 scans). Plot (b) shows results from fivefold replicates of samples neatly aligned in the laser focal point and with inconclusive results removed

As a next step, all 0–100% binary cocaine mixtures were analyzed, and the results shown on the device's screen were compared to the known sample composition (Table S1). Each of the 80 samples was scanned 10 times. For five of these scans, the sample was repeatedly placed in the instrument without any precautions to mimic practical measurements, and for five other scans, special attention was put in place to make sure samples were neatly aligned in front of the laser for optimal signal. For all samples, the test result shown on the screen was either “cocaine”, the actual identity of the present adulterant, or “inconclusive.” When the instrument returned the identity of the cutting agent for a cocaine‐containing sample, this was considered a FN result. Figure 1a shows the performance of the instrument on binary cocaine mixtures for all tenfold replicates and considering every non‐cocaine result (including inconclusive results) a FN. Figure S1 shows the comparison of these results with and without these inconclusive results. The latter gives a better representation of the actual forensic situation as an officer might perform a reanalysis after an inconclusive result. In general, inconclusive results were occasionally observed in scans performed without special attention for sample alignment in the focal point of the laser. These results were attributed to a less intense Raman signal not leading to a successful match by the built‐in library algorithms in the device. Figure 1b shows the results in the ideal situation of samples neatly aligned in the laser focal point and inconclusive results removed. When excluding the inconclusive results, the instrument correctly detected cocaine in all mixtures with 60 wt% cocaine HCl and above. Between 30 and 50 wt% cocaine, the majority of the cocaine‐containing mixtures were correctly identified; however, specific cutting agents yielded different LODs. For instance, procaine and paracetamol already showed a majority of FN results at 40 wt% cocaine levels; levamisole gave 60% FN results at 30 wt% cocaine, while cocaine mixed with inositol only remained undetected at 10 wt% cocaine. Although the exact mechanisms of the built‐in software are not disclosed, the operating procedures state that the system uses multiple hierarchical spectral libraries. Among other controlled substances, cocaine is included in the top priority library, and all cutting agents are included in a lower priority library. This means that the observed FNs resulted from both a nonmatch (below identification criteria) in the controlled substances library followed by a match in the nondrug library. A plausible explanation for the component‐specific LODs is that the Raman signal of cocaine may be obscured by various compounds following different mechanisms. Paracetamol is a fluorescent compound that is known for this effect, and the built‐in software provided a warning message that the presence of a narcotic cannot be ruled out.

#### 3.1.2 Performance on case material analyzed retrospectively

Table 2 shows the comparison of the GC–MS results and corresponding results provided by the handheld Raman analyzer's built‐in software for 3,168 case samples analyzed between 2015 and 2020. These results show a remarkably good 0% FP rate for cocaine, which is important as FP results can lead to wrongful judicial actions. It must however be noted that although no FP results were obtained for cocaine, incidental FP results were encountered for other controlled substances. Most notably, in five cases, the instrument reported the presence of fentanyl or a fentanyl‐derivate, whereas this compound was not observed with GC–MS. These samples were most often found to be amphetamine mixed with either MDMA, cocaine or adulterants. For 13% of the case samples, the instrument did not produce a result, showing “inconclusive” on its screen. The majority of these samples (174 out of 183) were found not to contain any cocaine based on the corresponding GC–MS results. Further assessment of the GC–MS data showed that these samples sometimes contained other drugs (e.g., MDMA and amphetamine), a cutting agent (levamisole) or—in most cases—did not show any identified peak in the GC–MS data. In 45 samples, the presence of cocaine was missed by the instrument leading to a FN (36×) or inconclusive (9×) result. These results predominantly originated from samples with a low cocaine content (estimated concentration by GC–MS below 30 wt%, average ~13 wt%) except six cocaine samples with an estimated cocaine content between 30 and 50 wt% that were adulterated with procaine, levamisole, and/or benzocaine. These findings are thus in line with the results shown in Figure 1. The relatively low percentage of FN results in this data set compared to the high number of FNs or inconclusive results for the binary mixtures described at Section 3.1.1 can be explained by a low prior probability to encounter low‐level cocaine samples in actual casework. FN results mainly occur for sub‐40 wt% cocaine‐content samples (Figure 1), but these are rarely encountered as the reported average cocaine content in street samples is around 60 wt%.^35^, ^36^

**TABLE 2:** Retrospective comparison of handheld Raman results compared with GC–MS results for 3,168 drug‐suspected case samples analyzed between 2015 and 2020 by the Amsterdam Police

GC–MS result	Handheld Raman result for cocaine		
	Positive	Negative	Inconclusive
Cocaine positive	1,778 (97.5%)	36 (2.0%)	9 (0.5%)
Cocaine negative	0 (0%)	1,390 (87.5%)	174 (12.5%)

Abbreviation: GC–MS, gas chromatography–mass spectrometry.

### 3.2 Spectral selectivity

**FIGURE 2:**
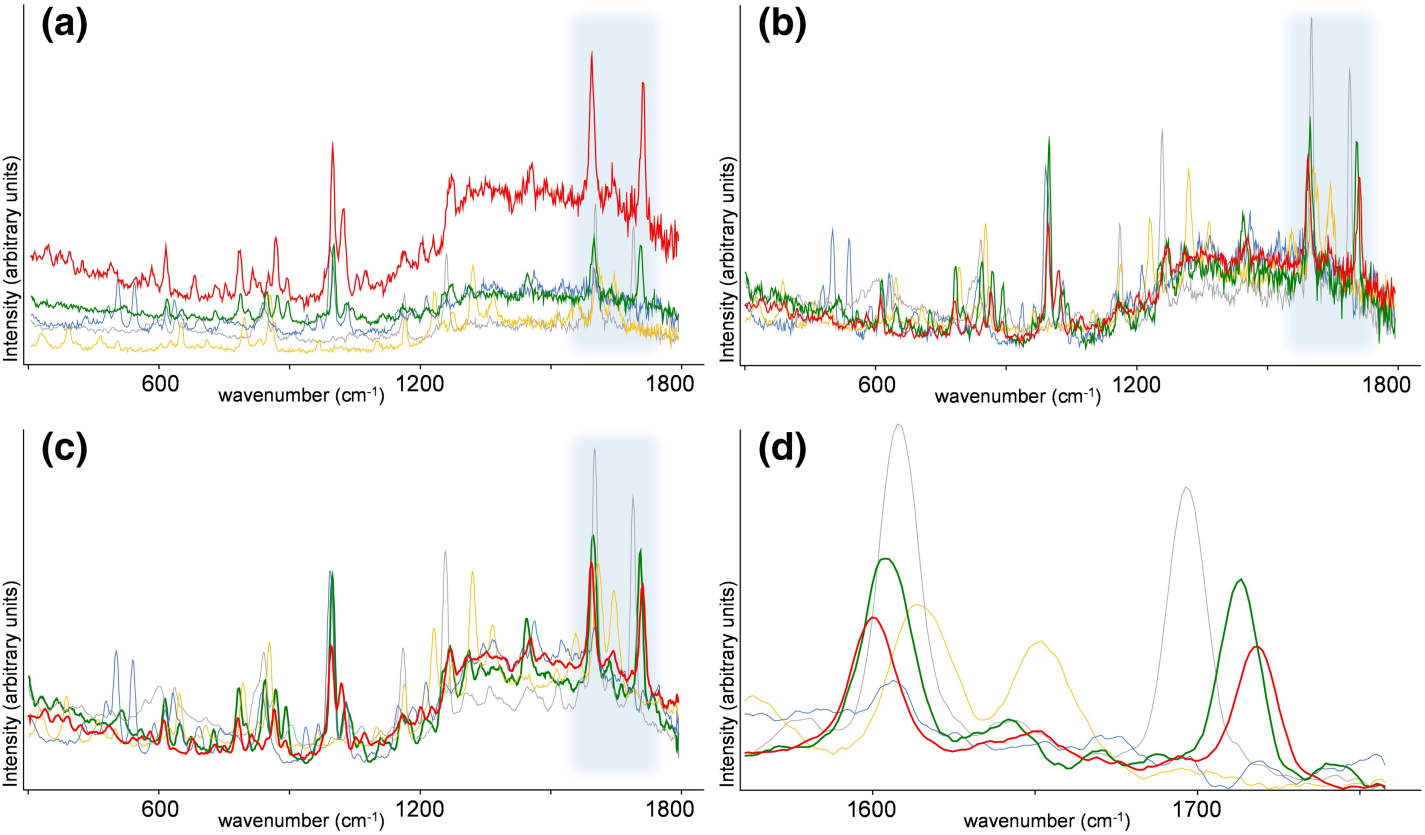
Raman spectra of cocaine and three cutting agents. (a) Raw spectral data; (b) after standard normal variate (SNV) preprocessing; (c) after subsequent Savitzky–Golay (SG) smoothing; (d) after 1,560‐ to 1,756‐cm^−1^ selection. Blue shaded areas indicate the spectral area of Panel (d). Spectra of cocaine HCl (red); cocaine base (green); procaine (gray); paracetamol (yellow); levamisole (blue)

To further investigate selectivity, the Raman spectral data were examined in more detail. Raw spectra showed major intensity differences both between replicate scans and among different substances. Although relative intensities of spectral peaks from a single compound were repeatable, large variation was observed in absolute intensity and baseline offset. Both are well‐known effects in direct spectroscopic analysis on solid samples caused by particle size differences, light scattering, and spectral interferences. Spectral preprocessing is a common strategy to extract useful information from the raw spectral data and remove nonselective systematic and random signal fluctuations.^24^, ^25^, ^37^ Figure 2 shows the (a) raw spectral data, (b) data after SNV preprocessing, (c) subsequent smoothing, and (d) a 1,560‐ to 1,756‐cm^−1^ selection with baseline correction for both cocaine HCl and cocaine base. Spectra of the cutting agents levamisole, paracetamol, and procaine are shown in the same plots to demonstrate the spectral selectivity. An example of the typical variation in raw spectral data and corresponding spectral consistency after SNV preprocessing can be found in the supplemental information (Figure S2). Full 300‐ to 1,800‐cm^−1^ Raman spectra of cocaine HCl, cocaine base and the eight most common cutting agents are shown in Figure S3. Around 1,000 cm^−1^, a spectral peak is present in both cocaine types and in levamisole. This spectral peak is absent in the other spectra in Figure S3. The 1,000 cm^−1^ Raman peak is associated with the aromatic ring breathing vibration, the symmetric stretching of the mono‐substituted aromatic ring,^14^ a moiety present in both cocaine and levamisole. However, cocaine could be easily distinguished from levamisole by the presence of a pair of spectral peaks at 1,599 cm^−1^ and 1,716 cm^−1^ for cocaine HCl or 1,603 and 1,712 cm^−1^ for cocaine base. These peaks originate from the C=C stretching in the aromatic ring (peak ~1,600 cm^−1^) and the symmetric stretching of the carbonyl group (peaks 1,712 and 1,716 cm^−1^) as proposed by Penido et al.^14^ The 1,560‐ to 1,756‐cm^−1^ spectral region around these two peaks was found highly diagnostic for cocaine as none of the substances included in this study yielded two peaks in this window at exactly the same position and relative intensity. Especially, the second peak in this region was selective as no single substance from all cutting agents, and other drug substances did show a prominent Raman signal above 1,700 cm^−1^. Common substances that did produce spectral features in this region are procaine, paracetamol, and phenacetin. As shown in Figure 2d, these three substances all yield a spectral peak near or even overlapping with the ~1,600‐cm^−1^ peak for cocaine. Procaine is the only substance in this study that had a spectral feature in close proximity—and slightly overlapping with—the ~1,700‐cm^−1^ cocaine peak. This high spectral selectivity explains the overall correct performance of the handheld Raman analyzer for high‐level cocaine samples. However, in mixtures, which is often the case in actual cocaine case samples,^38^, ^39^ the observed Raman spectrum originates from all present substances, and spectral bands can overlap or obscure each other. This will compromise traditional library searching strategies similarly as other direct spectroscopic analyses (such as FTIR) that are—without prior separation—not well suited for mixture analysis.^40^ As all substances yield both a different Raman signal intensity and different individual Raman peaks, it is evident that the instrument's performance, and detection range for cocaine varies per individual substance. The cutting agents that demonstrate the highest FN rate in the analyzer's built‐in software, namely procaine, paracetamol and (in a lesser extent) levamisole, and phenacetin are the same substances that show overlapping spectral peaks with at least one of the distinctive spectral features for cocaine. A single LOD for Raman‐based cocaine detection in powders thus cannot be determined as this is highly dependent on the sample composition.

### 3.3 Development of PLS‐based models

#### 3.3.1 Model design

PLS is a supervised chemometric approach requiring a binary variable for classification purposes (i.e., PLS‐DA) or a numerical value for quantification (i.e., PLS‐R). This study aims to develop a model to correctly classify case samples as either cocaine containing or cocaine negative. Therefore, PLS‐DA would be the method of choice if the majority of samples consisted of either pure cocaine or pure other (non‐cocaine) compounds. However, in this specific forensic situation, cocaine levels in street samples range between 40 wt% and 84 wt% with an average purity of around 60 wt%.^35^ The non‐cocaine part of these samples consists of many different cutting agents of which their occurrence may vary regionally.^36^, ^38^, ^39^, ^41^ In this light, spectra from cocaine‐positive samples may contain a majority of spectral signals that do not originate from cocaine itself and may negatively affect the performance of a classification model. For this reason, both a PLS‐R and a PLS‐DA model were developed with an emphasis on the most discriminating cocaine peaks in the 1,700–1,728 ROI area. Each model was optimized with preprocessing suiting the focus of the method (regression or classification). Although it could be argued that the confined ROI area with a small spectral window could be assessed using traditional univariate regression, preliminary results showed several erroneous results (e.g., procaine) and difficulties dealing with both cocaine HCl and cocaine base spectra. Therefore, a multivariate approach was applied to emphasize the contrast between the useful variation in the data and the noninformative data and noise. With multivariate approaches, sample dimensions are reduced, and the significant variation is transferred to latent variables. If such latent variables only describe the variation of the compound of interest, it becomes easier to discriminate between the target compound and interferants. This dimension reduction also facilitated outlier detection and resulted in an overall time‐efficient and convenient automated process.

**FIGURE 3:**
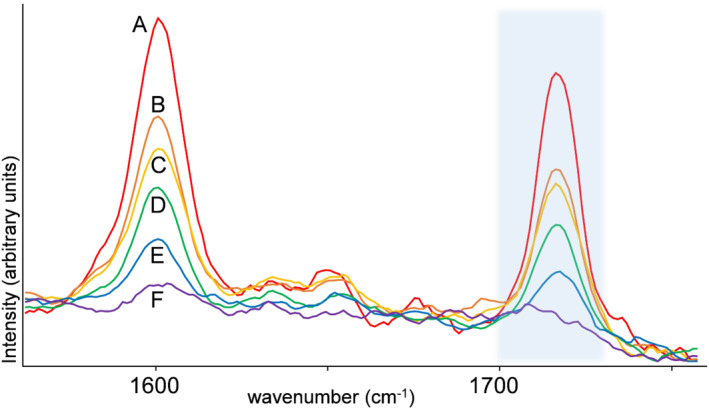
Cocaine selective Raman signals in the 1,560‐ to 1,756‐cm^−1^ region of the spectrum for cocaine–mannitol mixtures after standard normal variate (SNV) preprocessing on the full spectrum. (a) 90% cocaine HCl; (b) 70% cocaine HCl; (c) 50% cocaine HCl; (d) 30% cocaine HCl; (e) 20% cocaine HCl; (f) 10% cocaine HCl. Blue shade indicates the 1,700‐ to 1,728‐cm^−1^ region of interest (ROI) used for partial least squares (PLS) models

Firstly, a PLS‐R model was developed that predicted a concentration for unknown spectra. For a regression model, it is unlikely that a discrete 0.0% cocaine concentration is predicted for all non‐cocaine drugs and cutting agents. An adjustable cut‐off threshold on the predicted concentration is therefore applied in a similar fashion as earlier work using a near infrared (NIR)‐based model.^7^ In this way, the number of FP results can be reduced by accepting a certain degree of FN results for low‐level (i.e., below threshold) cocaine samples. From a legal point of view, even low‐level cocaine samples are controlled substances, and excluding these from subsequent further legal action is unwanted. This, however, might be acceptable because these samples are rarely encountered in case materials and often other techniques are also available, such as the generally more sensitive yet less selective colorimetric spot tests. Also, it must be noted that all handheld devices have a detection limit (which is often unknown and may vary between devices) and thus may lead to FN results for low‐level samples as was demonstrated in Section 3.1.1. Moreover, this threshold is acceptable if the handheld would solely be used for indicative purposes. The optimal signal preprocessing for PLS‐R was found to be SNV normalization on the full spectrum followed by ROI selection with a focus on the most selective cocaine peaks at 1,712 (HCl) and 1,716 cm^−1^ (base). ROI selection was performed after SNV to prevent erroneous results from samples that do not have any peaks in the ROI (e.g., non‐cocaine‐containing samples) , which could lead to excessive noise from the normalization of only minor spectral features. It must be noted that because a normalization step is involved in the spectral preprocessing, no linear correlations between absolute signal intensity and compound concentration are present. Quantitative results from the PLS‐R models are thus based on the relative combination of the spectral signals from the individual compounds in the sample. In the case that cocaine is mixed with a Raman‐transparent compound, a too high prediction can be encountered and vice versa for a cutting agent producing a strong Raman signal. Although other studies successfully developed a quantitative PLS‐R model after SNV,^26^, ^27^ our goal is not to develop a robust quantitative method. Predicted cocaine concentrations are only used for cocaine detection by comparing the predicted value against the threshold value to distinguish evident cocaine spectral features from possible noise. Figure 3 shows that concentration dependency is still visible after SNV‐preprocessing for mannitol mixtures as an example. This is explained by the presence of mannitol selective spectral features outside of the ROI area.

Additionally, a PLS‐DA model was developed similarly to the PLS‐R model. Contrary to PLS‐R, the preprocessing order was switched to first ROI selection and subsequent normalization (as described in Section 2.6.2) to put maximum emphasis on the presence of cocaine‐specific spectral features while eliminating all concentration dependency.

**FIGURE 4:**
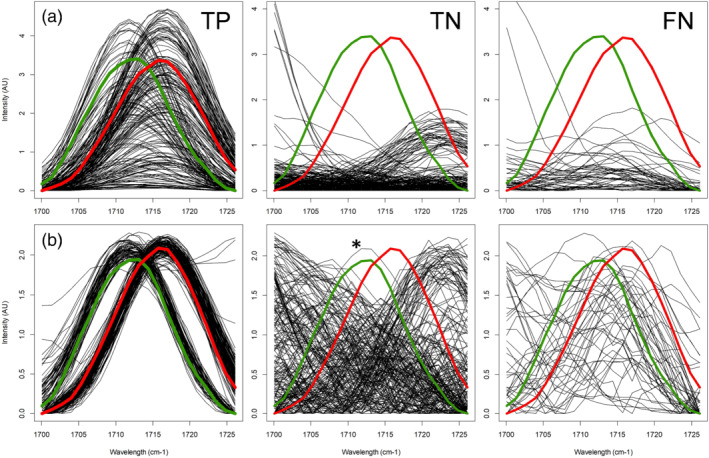
Cocaine specific Raman signals in the 1,700‐ to 1,728‐cm^−1^ region of interest (ROI) after (a) standard normal variate followed by region of interest selection (SNV‐ROI) preprocessing for partial least squares regression (PLS‐R) and (b) ROI selection followed by SNV preprocessing for partial least squares discriminant analysis (PLS‐DA). Overlay of cocaine HCl (red), cocaine base (green), and case samples from retrospective analysis (black). Two hundred true positive (TP) spectra, 200 true negative (TN) spectra, and 45 false negative (FN) spectra. * indicates one specific TN that was found to be inhomogeneous and containing a minimal amount of cocaine

The two preprocessing methods showed convincing spectral selectivity differences for cocaine containing versus negative samples as demonstrated in Figure 4. None of the negative samples showed a spectral peak in the ROI area following the preprocessing corresponding with the PLS‐R model (Figure 4a, panel TN). However, because concentration dependency is present, low‐level cocaine samples also yielded a less intense cocaine signal visible at the low‐intensity level of panel TP in Figure 4a. This can lead to some FNs when low‐level cocaine samples are predicted below a certain threshold value. On the other hand, all cocaine containing spectra visible in Figure 4b produce near‐similar spectral features after the preprocessing used for PLS‐DA from which they could easily be attributed to either cocaine base or cocaine HCl.

#### 3.3.2 Performance assessment

**FIGURE 5:**
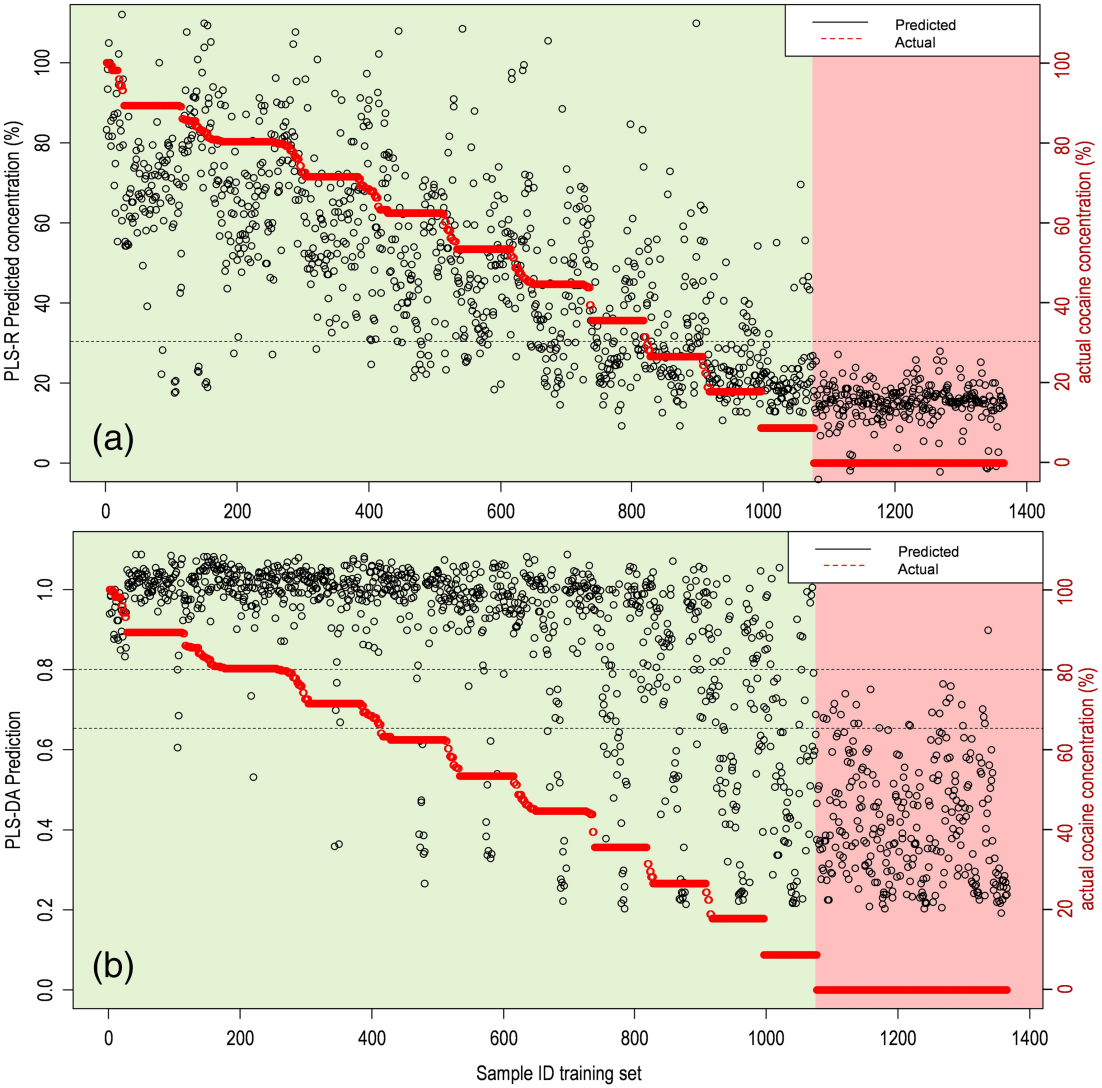
Predictions versus actual cocaine concentrations of the training set for the (a) partial least square regression (PLS‐R) and (b) discriminant analysis (PLS‐DA) model. Green panel indicates all cocaine‐containing samples; red panel indicates non‐cocaine samples. The black dotted lines indicate 30 wt%, 0.8 and 0.65 thresholds used for the combined identification criteria

Figure 5 shows the predicted versus actual cocaine levels for the training set of the model (results from cross‐validation). For the PLS‐R model (Figure 5a), all negative samples gave a predicted concentration below 30%, although many cocaine‐containing samples—especially at lower levels—were also predicted below this level. Contrary to this, the PLS‐DA model yielded much more variation for negative samples with prediction scores mainly between 0.2 and 0.8. This could be explained by the lack of cocaine‐specific spectral features resulting in noisy signals. Besides, cocaine containing samples that do show cocaine specific spectral features often produced a PLS‐DA prediction above 0.8 (Figure 5b).

As both developed PLS models have their strengths and weaknesses, results from both models were combined for optimal results. A prediction score equal to or higher than 0.8 in the PLS‐DA model proved a good indicator for cocaine‐containing samples. Also, a predicted cocaine concentration of 30 wt% or lower in the PLS‐R model was a strong indicator for the absence of cocaine. Although only limited negative samples showed a PLS‐R result between 20% and 30%, extending this threshold was not very effective as several cocaine‐containing samples were also predicted in this concentration range. The best result was obtained by applying the following detection criteria:

- If PLS‐DA prediction is ≥0.8: positive detection
- If PLS‐DA prediction is 0.65–0.8 and PLS‐R prediction is >30%: positive detection
- If PLS‐DA prediction is ≤0.65 or PLS‐R prediction is ≤30%: negative

In this way, only one out of 300 (0.3%) spectra of non‐cocaine samples in the training set was FP, and 76 out of 823 (9.2%) of the cocaine containing samples with a cocaine content above 30% were FN. These FNs were spectra in which cocaine specific spectral features were not visible in the ROI. These spectra in most cases also yielded FN results as obtained by the built‐in software (Section 3.1.1) indicating that spectral sensitivity is the limiting factor.

#### 3.3.3 Retrospective analysis of case material by PLS models

**FIGURE 6:**
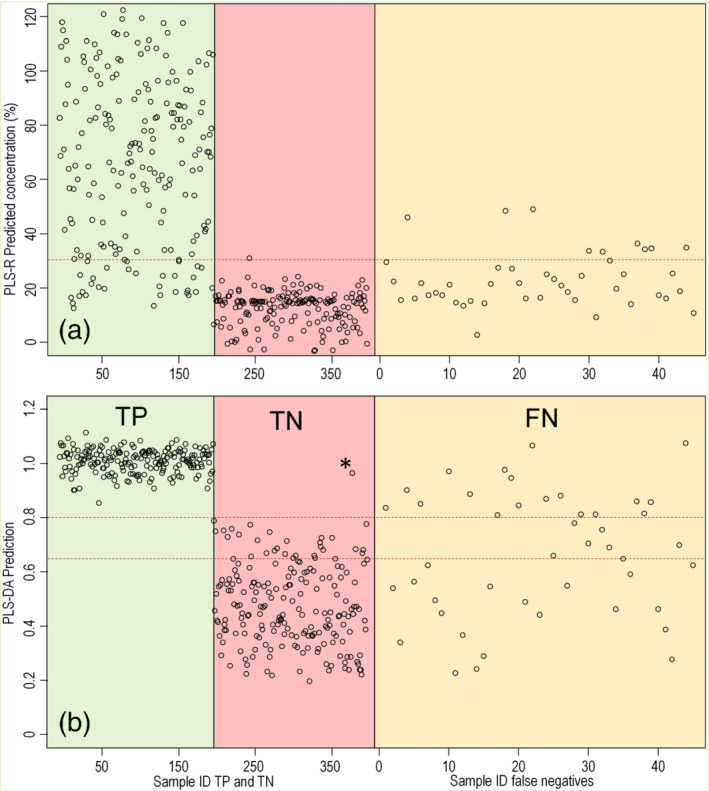
Predictions of Raman spectra from historic case samples (2015–2020) analyzed retrospectively by the (a) partial least square regression (PLS‐R) and (b) discriminant analysis (PLS‐DA) models. Samples 1–200 were reported as cocaine positive by both gas chromatography–mass spectrometry (GC–MS) and the Raman analyzer (true positive [TP], green shade); samples 201–400 were reported negative by both GC–MS and the Raman analyzer (true negative [TN], red shade); 45 cocaine‐containing samples were reported negative by the Raman analyzer's built‐in software (false negative [FN], orange shade). Red dotted lines indicate the identification criteria thresholds. * indicates one specific TN that was found to be inhomogeneous and containing a minimal amount of cocaine

Results of the Raman spectra from case samples analyzed between 2015 and 2020 (Section 2.5) were also predicted by the developed models. The predicted scores are shown in Figure 6. All 200 spectra from cocaine samples that were correctly identified by the built‐in software also were correctly predicted by the PLS‐R and PLS‐DA models using the combined detection criteria described in Section 3.3.2. Only one out of the 200 negative samples was falsely predicted as cocaine. This is visible as the only dot above the 0.8 threshold line for the TNs in Figure 6b. This particular sample, marked with an asterisk in both Figures 4b and 6b, was found to be an inhomogeneous case sample consisting of coarse grains with different shades of white color. Although this specific subsample was reported as containing levamisole, the original laboratory data from 2017 revealed that this sample was inhomogeneous and possibly contaminated with cocaine from other subsamples of the same seizure. GC–MS results of the homogenized sample showed a minor cocaine peak below the laboratory's reporting limit. A possible explanation for the presence of the cocaine‐specific Raman feature in the spectrum is that the focal point of the instrument's laser is less than 1 mm. In this way, the obtained Raman spectrum can originate from a single coarse particle that is not representative of the sample. Additionally, this result also indicates that chemometric analysis of the Raman spectral data can lead to a better sensitivity than observed with the analyzer's built‐in software. This is in line with PLS‐DA scores of the 10–80 wt% cocaine mixtures shown in Figure 5b where a majority of the samples were predicted with a >0.8 score indicating the presence of cocaine‐specific spectral features. However, such features were almost absent for samples with a sub‐0.5 score. The above 50 wt% cocaine samples that yielded a low score and lacked the cocaine‐specific peak in the spectrum were found to be the same as the FN or inconclusive results from the handheld spectrometer's built‐in software (Figure 1). Especially, in none of the spectra from 10–20 wt% cocaine mixtures with caffein, procaine or paracetamol, the cocaine‐specific features were visible. This again indicates that FN or inconclusive results from the handheld spectrometer on binary cocaine mixtures could more likely be attributed to spectral limitations or measurement errors than misidentification by the built‐in software.

**FIGURE 7:**
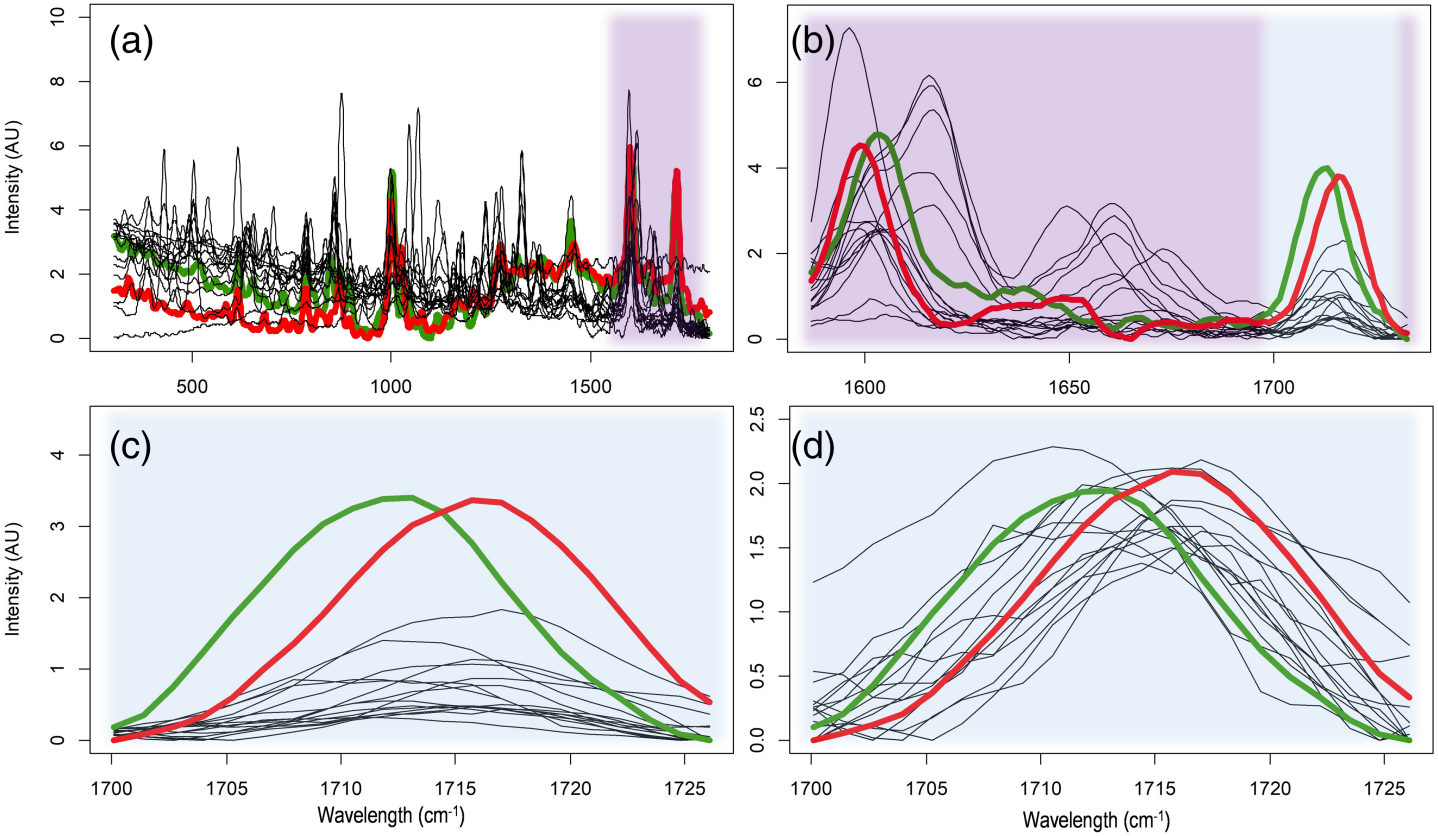
Raman spectra of cocaine‐containing case samples that were predicted false negative by the analyzer's built‐in software and predicted (true) positive by the combined partial least squares discriminant analysis (PLS‐DA) and regression (PLS‐R) model. (a) Full‐spectrum after standard normal variate (SNV); (b) zoom on the 1,560‐ to 1,756‐cm^−1^ spectral area containing two cocaine‐specific peaks; (c) the region of interest (ROI) area after the preprocessing used for PLS‐R model: SNV followed by ROI; (d) spectra after PLS‐DA preprocessing: ROI followed by SNV. All spectra overlaid with reference spectra of cocaine HCl (red) and cocaine base (green)

The 45 spectra reported as negative or inconclusive by the handheld Raman analyzer although cocaine was identified by GC–MS (set FN from retrospective analysis) were also assessed by the PLS‐models. In this way, 21 of these spectra retrospectively resulted in a positive result for the presence of cocaine. Raman spectra of these samples showed a peak at either 1,712 or 1,716 cm^−1^ visible in the normalized ROI area as shown in Figure 7d. These peaks were also visible in this ROI area when the SNV normalization was applied on the entire spectrum, albeit at lower intensity due to strong Raman signals in the spectrum that do not originate from cocaine (Figure 7a–c). The 1,712‐ (base) and 1,716‐cm^−1^ (HCl) features were again found to be the only selective peaks for cocaine as other spectral reference peaks were completely or partly obscured by adulterants present in the samples (e.g., peaks 1,599 cm^−1^ for cocaine HCl and 1,603 cm^−1^ for cocaine base as visible in Figure 7b). The high selectivity of the 1,712‐ and 1,716‐cm^−1^ peaks is in line with the TNs in Figure 4 where none of the large variety of non‐cocaine case samples showed a peak in this area. This indicates that these peaks, attributed to the symmetric stretching of the C=O bond in the carbonyl group of the cocaine molecule^14^ can be used as a specific and sensitive indicator for cocaine presence when analyzing case samples with Raman spectroscopy. A further improvement of the detectability at lower concentrations could possibly be achieved by more advanced chemometric or machine learning models with focus on a wider range of cocaine‐specific Raman signals in the full spectrum. However, this could increase the risk of FP results and might not be suitable for samples where the limiting factor is of spectroscopic nature and Raman peaks of cocaine are absent in the spectrum due to, for example, absorption of the laser light or strong fluorescence.

### 3.4 Discussion

This work demonstrates that the LOD for cocaine detected by a handheld Raman analyzer is highly dependent of sample composition and can vary between 40 wt% for cocaine samples containing procaine or paracetamol to 10 wt% for cocaine samples containing inositol. Although a worst‐case LOD of near 40 wt% seems rather problematic, the composition of typical cocaine‐containing street samples justifies its application in a forensic setting. The prior probability to encounter a sub‐40 wt% cocaine sample in forensic cases is very low because the reported cocaine content in street samples in Europe is often well above this level with averages up to 70 wt%.^35^, ^36^ This is in line with the results from the retrospective analysis described in Section 3.1.2 where only 2.5% of the cocaine‐containing case samples analyzed between 2015 and 2020 were missed by the instrument's built‐in detection algorithms. In addition, the absence of FP results for cocaine within these 3,168 samples furthermore underlines its applicability as a reliable cocaine detector in forensic casework. The results show that when cocaine is reported by the instrument's built‐in software, it is very unlikely that this is a FP result and that the sample does not contain cocaine. In this way, additional spectral assessment and confirmation of the presence of cocaine specific spectral features such as demonstrated in this study may fulfill the SWGDRUG requirements for unambiguous cocaine identification eliminating the need for subsequent GC–MS analysis for certain highly concentrated cocaine samples. On the other hand, when no cocaine or other drug is reported, it is plausible that a controlled substance is still present. It must thus be noted that this study only focuses on cocaine detection. Other controlled substances may produce a less abundant or less selective Raman spectrum leading to different TP and TN percentages in forensic casework. The reported figures are also only valid for light‐colored solid samples (e.g., powders, chunks, and compressed powders) seized in a drug‐suspected forensic setting. Common other drugs of abuse such as heroin and MDMA are often seized as brown powders or in colored tablets respectively, and both these substances yield fluorescence. LODs using direct Raman spectroscopy are therefore expected to be high, and the use of dedicated SERS kits is suggested to detect these compounds. In general, colored samples, samples containing highly fluorescent substances or samples with a higher prior probability to contain a lower concentration of cocaine, can lead to worse figures.

## 4 CONCLUSIONS

The TruNarc handheld Raman analyzer is suitable for reliable cocaine detection in forensic case samples. Detection limits were found to be highly dependent on the type of adulterants present in the sample. For cocaine mixed with procaine, paracetamol or caffeine LODs around 30–40 wt% were found. Because the average cocaine content in seized case samples in Europe often exceeds 50 wt%,^35^ the instrument could still be successfully applied for on‐scene cocaine detection in this specific forensic setting. After comparing GC–MS and Raman data of 3,168 case samples analyzed between 2015 and 2020, 97.5% TP and 87.5% TN were determined. No FP results were obtained with the instrument, although 12.5% of the non‐cocaine samples were reported as being inconclusive. The spectral selectivity allowing for unambiguous cocaine detection was confirmed by the presence of several cocaine‐specific spectral features. Especially the 1,712‐ and 1,716‐cm^−1^ Raman peaks were found highly selective as, besides cocaine, none of the drugs‐of‐abuse, cutting agents or pharmaceuticals included in this study yielded a spectral peak at these wavenumbers. A dual‐stage PLS‐DA and PLS‐R model with an emphasis on these two peaks further increased the performance by correctly predicting 21 of the 45 cocaine containing samples that were previously missed by the instrument. Overall, these results demonstrate that reliable on‐scene cocaine detection is feasible using the TruNarc handheld Raman analyzer and its built‐in software. Additionally, the instrument's spectral resolution and selectivity for cocaine was found adequate for incorporation in analytical schemes ultimately leading to court evidence from handheld techniques.

## CONFLICT OF INTEREST

The authors declare no competing interest.

## AUTHOR CONTRIBUTION

Ruben F. Kranenburg: conceptualization, methodology, investigation, formal analysis, data curation, and writing – original draft; Joshka Verduin: investigation, methodology, and writing – review & editing; Renee de Ridder: investigation; Yannick Weesepoel: methodology and writing – review & editing; Martin Alewijn: software, methodology, and writing – review & editing; Marcel Heerschop: investigation; Peter Keizers: writing – review & editing; Annette van Esch: investigation; Arian C. van Asten: supervision and writing – review & editing.

## Supporting information

Table S1. Results TruNarc scans for diluted cocaine samples.
Table S2. Results TruNarc scans for drugs‐of‐abuse compounds, cutting agents and non‐cocaine mixtures.
Table S3. Results TruNarc scans for cocaine‐containing case samples.
Figure S1. False‐negative results of the TruNarc handheld Raman spectrometer for binary cocaine mixtures with 8 common cutting agents at concentrations ranging from 10–100 wt% cocaine.
Figure S2. Raman spectra of 5 replicate scans of a single cocaine HCl sample.
Figure S3. Raman spectra of cocaine and common cutting agents.
