# Supplementary material for: Performance evaluation of handheld Raman spectroscopy for cocaine detection in forensic case samples
Source: Drug Test Anal. 2021 Jan 7;13(5):1054–67. doi: 10.1002/dta.2993 (PMC8248000; doi:10.1002/dta.2993)
Supplement: Supplementary file 1 — Table S1. Results TruNarc scans for diluted cocaine samples. Table S2. Results TruNarc scans for drugs‐of‐abuse compounds, cutting agents and non‐cocaine mixtures. Table S3. Results TruNarc scans for cocaine‐containing case samples. Figure S1. False‐negative results of the TruNarc handheld Raman spectrometer for binary cocaine mixtures with 8 common cutting agents at concentrations ranging from 10–100 wt% cocaine. Figure S2. Raman spectra of 5 replicate scans of a single cocaine HCl sample. Figure S3. Raman spectra of cocaine and common cutting agents. [file DTA-13-1054-s001.pdf]

Supplemental Information

for

Performance Evaluation of Handheld Raman Spectroscopy for Cocaine Detection in Forensic Case Samples

Ruben F. Kranenburg[1,2,*], Joshka Verduin[1,2], Renee de Ridder[1], Yannick Weesepoel[3], Martin Alewijn[3], Marcel Heerschop[4], Peter Keizers[5], Annette van Esch[6], Arian C. van Asten[2,7]

[1] Dutch National Police, Unit Amsterdam, Forensic Laboratory, Kabelweg 25, Amsterdam 1014 BA, The Netherlands

[2] Van 't Hoff Institute for Molecular Sciences, University of Amsterdam, Postbus 94157, Amsterdam 1090 GD, The Netherlands

[3] Wageningen Food Safety Research part of Wageningen University and Research, Akkermaalsbos 2, Wageningen 6708 WB, The Netherlands

[4] Dutch Customs Laboratory, Kingsfordweg 1, Amsterdam 1043 GN, The Netherlands

[5] National Institute of Public Health and the Environment (RIVM), Antonie van Leeuwenhoeklaan 9, Bilthoven 3721 MA, The Netherlands

[6] Netherlands Forensic Institute (NFI), Laan van Ypenburg 6, Den Haag 2497 GB, The Netherlands

[7] Co van Ledden Hulsebosch Center (CLHC), Amsterdam Center for Forensic Science and Medicine, Postbus 94157, Amsterdam 1090 GD, The Netherlands

[*] Corresponding author. *E-mail address:* ruben.kranenburg@politie.nl (R.F. Kranenburg).

Table S1. Results TruNarc scans for diluted cocaine samples.
Table S2. Results TruNarc scans for drugs-of-abuse compounds, cutting agents and non-cocaine mixtures.
Table S3. Results TruNarc scans for cocaine-containing case samples.
Figure S1. False-negative results of the TruNarc handheld Raman spectrometer for binary cocaine mixtures with 8 common cutting-agents at concentrations ranging from 10 – 100 wt% cocaine.
Figure S2. Raman spectra of 5 replicate scans of a single cocaine HCl sample.
Figure S3. Raman spectra of cocaine and common cutting agents.

Table S1. Results TruNarc scans for diluted cocaine samples.

scan	composition	Raman result	type
DIL1-1	cocaine: caffeine, 0 : 100	caffeine	TN
DIL1-2	cocaine: caffeine, 0 : 100	caffeine	TN
DIL1-3	cocaine: caffeine, 0 : 100	caffeine	TN
DIL1-4	cocaine: caffeine, 0 : 100	caffeine	TN
DIL1-5	cocaine: caffeine, 0 : 100	caffeine	TN
DIL1-6	cocaine: caffeine, 0 : 100	caffeine	TN
DIL1-7	cocaine: caffeine, 0 : 100	caffeine	TN
DIL1-8	cocaine: caffeine, 0 : 100	caffeine	TN
DIL1-9	cocaine: caffeine, 0 : 100	caffeine	TN
DIL1-10	cocaine: caffeine, 0 : 100	caffeine	TN
DIL2-1	cocaine: caffeine, 10 : 90	caffeine	FN
DIL2-2	cocaine: caffeine, 10 : 90	caffeine	FN
DIL2-3	cocaine: caffeine, 10 : 90	caffeine	FN
DIL2-4	cocaine: caffeine, 10 : 90	caffeine	FN
DIL2-5	cocaine: caffeine, 10 : 90	caffeine	FN
DIL2-6	cocaine: caffeine, 10 : 90	caffeine	FN
DIL2-7	cocaine: caffeine, 10 : 90	caffeine	FN
DIL2-8	cocaine: caffeine, 10 : 90	caffeine	FN
DIL2-9	cocaine: caffeine, 10 : 90	caffeine	FN
DIL2-10	cocaine: caffeine, 10 : 90	caffeine	FN
DIL3-1	cocaine: caffeine, 20 : 80	caffeine	FN
DIL3-2	cocaine: caffeine, 20 : 80	caffeine	FN
DIL3-3	cocaine: caffeine, 20 : 80	caffeine	FN
DIL3-4	cocaine: caffeine, 20 : 80	caffeine	FN
DIL3-5	cocaine: caffeine, 20 : 80	cocaine HCl	TP
DIL3-6	cocaine: caffeine, 20 : 80	caffeine	FN
DIL3-7	cocaine: caffeine, 20 : 80	caffeine	FN
DIL3-8	cocaine: caffeine, 20 : 80	caffeine	FN
DIL3-9	cocaine: caffeine, 20 : 80	caffeine	FN
DIL3-10	cocaine: caffeine, 20 : 80	caffeine	FN
DIL4-1	cocaine: caffeine, 30 : 70	caffeine	FN
DIL4-2	cocaine: caffeine, 30 : 70	caffeine	FN
DIL4-3	cocaine: caffeine, 30 : 70	caffeine	FN
DIL4-4	cocaine: caffeine, 30 : 70	caffeine	FN
DIL4-5	cocaine: caffeine, 30 : 70	caffeine	FN
DIL4-6	cocaine: caffeine, 30 : 70	caffeine	FN
DIL4-7	cocaine: caffeine, 30 : 70	cocaine HCl	TP
DIL4-8	cocaine: caffeine, 30 : 70	caffeine	FN
DIL4-9	cocaine: caffeine, 30 : 70	caffeine	FN
DIL4-10	cocaine: caffeine, 30 : 70	caffeine	FN
DIL5-1	cocaine: caffeine, 40 : 60	cocaine HCl	TP
DIL5-2	cocaine: caffeine, 40 : 60	cocaine HCl	TP
DIL5-3	cocaine: caffeine, 40 : 60	cocaine HCl	TP
DIL5-4	cocaine: caffeine, 40 : 60	cocaine HCl	TP
DIL5-5	cocaine: caffeine, 40 : 60	caffeine	FN
DIL5-6	cocaine: caffeine, 40 : 60	cocaine HCl	TP
DIL5-7	cocaine: caffeine, 40 : 60	caffeine	FN
DIL5-8	cocaine: caffeine, 40 : 60	cocaine HCl	TP
DIL5-9	cocaine: caffeine, 40 : 60	caffeine	FN
DIL5-10	cocaine: caffeine, 40 : 60	caffeine	FN
DIL6-1	cocaine: caffeine, 50 : 50	cocaine HCl	TP
DIL6-2	cocaine: caffeine, 50 : 50	caffeine	FN
DIL6-3	cocaine: caffeine, 50 : 50	cocaine HCl	TP
DIL6-4	cocaine: caffeine, 50 : 50	cocaine HCl	TP
DIL6-5	cocaine: caffeine, 50 : 50	cocaine HCl	TP
DIL6-6	cocaine: caffeine, 50 : 50	cocaine HCl	TP
DIL6-7	cocaine: caffeine, 50 : 50	caffeine	FN
DIL6-8	cocaine: caffeine, 50 : 50	cocaine HCl	TP
DIL6-9	cocaine: caffeine, 50 : 50	caffeine	FN
DIL6-10	cocaine: caffeine, 50 : 50	cocaine HCl	TP
DIL7-1	cocaine: caffeine, 60 : 40	cocaine HCl	TP
DIL7-2	cocaine: caffeine, 60 : 40	cocaine HCl	TP
DIL7-3	cocaine: caffeine, 60 : 40	cocaine HCl	TP
DIL7-4	cocaine: caffeine, 60 : 40	cocaine HCl	TP
DIL7-5	cocaine: caffeine, 60 : 40	cocaine HCl	TP
DIL7-6	cocaine: caffeine, 60 : 40	cocaine HCl	TP
DIL7-7	cocaine: caffeine, 60 : 40	cocaine HCl	TP
DIL7-8	cocaine: caffeine, 60 : 40	cocaine HCl	TP
DIL7-9	cocaine: caffeine, 60 : 40	cocaine HCl	TP
DIL7-10	cocaine: caffeine, 60 : 40	cocaine HCl	TP
DIL8-1	cocaine: caffeine, 70 : 30	cocaine HCl	TP
DIL8-2	cocaine: caffeine, 70 : 30	cocaine HCl	TP
DIL8-3	cocaine: caffeine, 70 : 30	cocaine HCl	TP

scan	composition	Raman result	type
DIL45-1	cocaine: procaine, 0 : 100	procaine	TN
DIL45-2	cocaine: procaine, 0 : 100	procaine	TN
DIL45-3	cocaine: procaine, 0 : 100	procaine	TN
DIL45-4	cocaine: procaine, 0 : 100	procaine	TN
DIL45-5	cocaine: procaine, 0 : 100	procaine	TN
DIL45-6	cocaine: procaine, 0 : 100	procaine	TN
DIL45-7	cocaine: procaine, 0 : 100	procaine	TN
DIL45-8	cocaine: procaine, 0 : 100	procaine	TN
DIL45-9	cocaine: procaine, 0 : 100	procaine	TN
DIL45-10	cocaine: procaine, 0 : 100	procaine	TN
DIL46-1	cocaine: procaine, 10 : 90	procaine	FN
DIL46-2	cocaine: procaine, 10 : 90	procaine	FN
DIL46-3	cocaine: procaine, 10 : 90	procaine	FN
DIL46-4	cocaine: procaine, 10 : 90	procaine	FN
DIL46-5	cocaine: procaine, 10 : 90	procaine	FN
DIL46-6	cocaine: procaine, 10 : 90	procaine	FN
DIL46-7	cocaine: procaine, 10 : 90	procaine	FN
DIL46-8	cocaine: procaine, 10 : 90	procaine	FN
DIL46-9	cocaine: procaine, 10 : 90	procaine	FN
DIL46-10	cocaine: procaine, 10 : 90	procaine	FN
DIL47-1	cocaine: procaine, 20 : 80	procaine	FN
DIL47-2	cocaine: procaine, 20 : 80	procaine	FN
DIL47-3	cocaine: procaine, 20 : 80	procaine	FN
DIL47-4	cocaine: procaine, 20 : 80	procaine	FN
DIL47-5	cocaine: procaine, 20 : 80	cocaine HCl	TP
DIL47-6	cocaine: procaine, 20 : 80	procaine	FN
DIL47-7	cocaine: procaine, 20 : 80	cocaine HCl	TP
DIL47-8	cocaine: procaine, 20 : 80	procaine	FN
DIL47-9	cocaine: procaine, 20 : 80	procaine	FN
DIL47-10	cocaine: procaine, 20 : 80	procaine	FN
DIL48-1	cocaine: procaine, 30 : 70	procaine	FN
DIL48-2	cocaine: procaine, 30 : 70	procaine	FN
DIL48-3	cocaine: procaine, 30 : 70	cocaine HCl	TP
DIL48-4	cocaine: procaine, 30 : 70	procaine	FN
DIL48-5	cocaine: procaine, 30 : 70	procaine	FN
DIL48-6	cocaine: procaine, 30 : 70	procaine	FN
DIL48-7	cocaine: procaine, 30 : 70	procaine	FN
DIL48-8	cocaine: procaine, 30 : 70	cocaine HCl	TP
DIL48-9	cocaine: procaine, 30 : 70	procaine	FN
DIL48-10	cocaine: procaine, 30 : 70	procaine	FN
DIL49-1	cocaine: procaine, 40 : 60	cocaine HCl	TP
DIL49-2	cocaine: procaine, 40 : 60	procaine	FN
DIL49-3	cocaine: procaine, 40 : 60	cocaine HCl	TP
DIL49-4	cocaine: procaine, 40 : 60	procaine	FN
DIL49-5	cocaine: procaine, 40 : 60	procaine	FN
DIL49-6	cocaine: procaine, 40 : 60	procaine	FN
DIL49-7	cocaine: procaine, 40 : 60	procaine	FN
DIL49-8	cocaine: procaine, 40 : 60	cocaine HCl	TP
DIL49-9	cocaine: procaine, 40 : 60	cocaine HCl	TP
DIL49-10	cocaine: procaine, 40 : 60	cocaine HCl	TP
DIL50-1	cocaine: procaine, 50 : 50	procaine	FN
DIL50-2	cocaine: procaine, 50 : 50	cocaine HCl	TP
DIL50-3	cocaine: procaine, 50 : 50	cocaine HCl	TP
DIL50-4	cocaine: procaine, 50 : 50	procaine	FN
DIL50-5	cocaine: procaine, 50 : 50	cocaine HCl	TP
DIL50-6	cocaine: procaine, 50 : 50	procaine	FN
DIL50-7	cocaine: procaine, 50 : 50	cocaine HCl	TP
DIL50-8	cocaine: procaine, 50 : 50	cocaine HCl	TP
DIL50-9	cocaine: procaine, 50 : 50	procaine	FN
DIL50-10	cocaine: procaine, 50 : 50	cocaine HCl	TP
DIL51-1	cocaine: procaine, 60 : 40	cocaine HCl	TP
DIL51-2	cocaine: procaine, 60 : 40	cocaine HCl	TP
DIL51-3	cocaine: procaine, 60 : 40	cocaine HCl	TP
DIL51-4	cocaine: procaine, 60 : 40	cocaine HCl	TP
DIL51-5	cocaine: procaine, 60 : 40	cocaine HCl	TP
DIL51-6	cocaine: procaine, 60 : 40	cocaine HCl	TP
DIL51-7	cocaine: procaine, 60 : 40	cocaine HCl	TP
DIL51-8	cocaine: procaine, 60 : 40	cocaine HCl	TP
DIL51-9	cocaine: procaine, 60 : 40	cocaine HCl	TP
DIL51-10	cocaine: procaine, 60 : 40	cocaine HCl	TP
DIL52-1	cocaine: procaine, 70 : 30	cocaine HCl	TP
DIL52-2	cocaine: procaine, 70 : 30	cocaine HCl	TP
DIL52-3	cocaine: procaine, 70 : 30	cocaine HCl	TP

DIL8-4	cocaine: caffeine, 70 : 30	cocaine HCl	TP
DIL8-5	cocaine: caffeine, 70 : 30	cocaine HCl	TP
DIL8-6	cocaine: caffeine, 70 : 30	cocaine HCl	TP
DIL8-7	cocaine: caffeine, 70 : 30	inconclusive	inconclusive
DIL8-8	cocaine: caffeine, 70 : 30	cocaine HCl	TP
DIL8-9	cocaine: caffeine, 70 : 30	cocaine HCl	TP
DIL8-10	cocaine: caffeine, 70 : 30	cocaine HCl	TP
DIL9-1	cocaine: caffeine, 80 : 20	cocaine HCl	TP
DIL9-2	cocaine: caffeine, 80 : 20	cocaine HCl	TP
DIL9-3	cocaine: caffeine, 80 : 20	cocaine HCl	TP
DIL9-4	cocaine: caffeine, 80 : 20	cocaine HCl	TP
DIL9-5	cocaine: caffeine, 80 : 20	cocaine HCl	TP
DIL9-6	cocaine: caffeine, 80 : 20	cocaine HCl	TP
DIL9-7	cocaine: caffeine, 80 : 20	cocaine HCl	TP
DIL9-8	cocaine: caffeine, 80 : 20	cocaine HCl	TP
DIL9-9	cocaine: caffeine, 80 : 20	cocaine HCl	TP
DIL9-10	cocaine: caffeine, 80 : 20	cocaine HCl	TP
DIL10-1	cocaine: caffeine, 90 : 10	cocaine HCl	TP
DIL10-2	cocaine: caffeine, 90 : 10	cocaine HCl	TP
DIL10-3	cocaine: caffeine, 90 : 10	cocaine HCl	TP
DIL10-4	cocaine: caffeine, 90 : 10	cocaine HCl	TP
DIL10-5	cocaine: caffeine, 90 : 10	cocaine HCl	TP
DIL10-6	cocaine: caffeine, 90 : 10	cocaine HCl	TP
DIL10-7	cocaine: caffeine, 90 : 10	cocaine HCl	TP
DIL10-8	cocaine: caffeine, 90 : 10	cocaine HCl	TP
DIL10-9	cocaine: caffeine, 90 : 10	cocaine HCl	TP
DIL10-10	cocaine: caffeine, 90 : 10	cocaine HCl	TP
DIL11-1	cocaine: caffeine, 100 : 0	cocaine HCl	TP
DIL11-2	cocaine: caffeine, 100 : 0	cocaine HCl	TP
DIL11-3	cocaine: caffeine, 100 : 0	cocaine HCl	TP
DIL11-4	cocaine: caffeine, 100 : 0	cocaine HCl	TP
DIL11-5	cocaine: caffeine, 100 : 0	cocaine HCl	TP
DIL11-6	cocaine: caffeine, 100 : 0	cocaine HCl	TP
DIL11-7	cocaine: caffeine, 100 : 0	cocaine HCl	TP
DIL11-8	cocaine: caffeine, 100 : 0	cocaine HCl	TP
DIL11-9	cocaine: caffeine, 100 : 0	cocaine HCl	TP
DIL11-10	cocaine: caffeine, 100 : 0	cocaine HCl	TP
DIL12-1	cocaine: lidocaine, 0 : 100	lidocaine	TN
DIL12-2	cocaine: lidocaine, 0 : 100	lidocaine	TN
DIL12-3	cocaine: lidocaine, 0 : 100	lidocaine	TN
DIL12-4	cocaine: lidocaine, 0 : 100	lidocaine	TN
DIL12-5	cocaine: lidocaine, 0 : 100	lidocaine	TN
DIL12-6	cocaine: lidocaine, 0 : 100	lidocaine	TN
DIL12-7	cocaine: lidocaine, 0 : 100	lidocaine	TN
DIL12-8	cocaine: lidocaine, 0 : 100	lidocaine	TN
DIL12-9	cocaine: lidocaine, 0 : 100	lidocaine	TN
DIL12-10	cocaine: lidocaine, 0 : 100	lidocaine	TN
DIL13-1	cocaine: lidocaine, 10 : 90	lidocaine	FN
DIL13-2	cocaine: lidocaine, 10 : 90	lidocaine	FN
DIL13-3	cocaine: lidocaine, 10 : 90	lidocaine	FN
DIL13-4	cocaine: lidocaine, 10 : 90	lidocaine	FN
DIL13-5	cocaine: lidocaine, 10 : 90	lidocaine	FN
DIL13-6	cocaine: lidocaine, 10 : 90	cocaine HCl	TP
DIL13-7	cocaine: lidocaine, 10 : 90	lidocaine	FN
DIL13-8	cocaine: lidocaine, 10 : 90	cocaine HCl	TP
DIL13-9	cocaine: lidocaine, 10 : 90	lidocaine	FN
DIL13-10	cocaine: lidocaine, 10 : 90	lidocaine	FN
DIL14-1	cocaine: lidocaine, 20 : 80	lidocaine	FN
DIL14-2	cocaine: lidocaine, 20 : 80	lidocaine	FN
DIL14-3	cocaine: lidocaine, 20 : 80	cocaine HCl	TP
DIL14-4	cocaine: lidocaine, 20 : 80	cocaine HCl	TP
DIL14-5	cocaine: lidocaine, 20 : 80	lidocaine	FN
DIL14-6	cocaine: lidocaine, 20 : 80	lidocaine	FN
DIL14-7	cocaine: lidocaine, 20 : 80	lidocaine	FN
DIL14-8	cocaine: lidocaine, 20 : 80	cocaine HCl	TP
DIL14-9	cocaine: lidocaine, 20 : 80	lidocaine	FN
DIL14-10	cocaine: lidocaine, 20 : 80	cocaine HCl	TP
DIL15-1	cocaine: lidocaine, 30 : 70	lidocaine	FN
DIL15-2	cocaine: lidocaine, 30 : 70	cocaine HCl	TP
DIL15-3	cocaine: lidocaine, 30 : 70	cocaine HCl	TP
DIL15-4	cocaine: lidocaine, 30 : 70	cocaine HCl	TP
DIL15-5	cocaine: lidocaine, 30 : 70	cocaine HCl	TP
DIL15-6	cocaine: lidocaine, 30 : 70	cocaine HCl	TP
DIL15-7	cocaine: lidocaine, 30 : 70	cocaine HCl	TP
DIL15-8	cocaine: lidocaine, 30 : 70	lidocaine	FN
DIL15-9	cocaine: lidocaine, 30 : 70	lidocaine	FN

DIL52-4	cocaine: procaine, 70 : 30	cocaine HCl	TP
DIL52-5	cocaine: procaine, 70 : 30	cocaine HCl	TP
DIL52-6	cocaine: procaine, 70 : 30	cocaine HCl	TP
DIL52-7	cocaine: procaine, 70 : 30	cocaine HCl	TP
DIL52-8	cocaine: procaine, 70 : 30	cocaine HCl	TP
DIL52-9	cocaine: procaine, 70 : 30	cocaine HCl	TP
DIL52-10	cocaine: procaine, 70 : 30	cocaine HCl	TP
DIL53-1	cocaine: procaine, 80 : 20	cocaine HCl	TP
DIL53-2	cocaine: procaine, 80 : 20	cocaine HCl	TP
DIL53-3	cocaine: procaine, 80 : 20	cocaine HCl	TP
DIL53-4	cocaine: procaine, 80 : 20	cocaine HCl	TP
DIL53-5	cocaine: procaine, 80 : 20	cocaine HCl	TP
DIL53-6	cocaine: procaine, 80 : 20	cocaine HCl	TP
DIL53-7	cocaine: procaine, 80 : 20	cocaine HCl	TP
DIL53-8	cocaine: procaine, 80 : 20	cocaine HCl	TP
DIL53-9	cocaine: procaine, 80 : 20	cocaine HCl	TP
DIL53-10	cocaine: procaine, 80 : 20	cocaine HCl	TP
DIL54-1	cocaine: procaine, 90 : 10	cocaine HCl	TP
DIL54-2	cocaine: procaine, 90 : 10	cocaine HCl	TP
DIL54-3	cocaine: procaine, 90 : 10	cocaine HCl	TP
DIL54-4	cocaine: procaine, 90 : 10	cocaine HCl	TP
DIL54-5	cocaine: procaine, 90 : 10	cocaine HCl	TP
DIL54-6	cocaine: procaine, 90 : 10	cocaine HCl	TP
DIL54-7	cocaine: procaine, 90 : 10	cocaine HCl	TP
DIL54-8	cocaine: procaine, 90 : 10	cocaine HCl	TP
DIL54-9	cocaine: procaine, 90 : 10	cocaine HCl	TP
DIL54-10	cocaine: procaine, 90 : 10	cocaine HCl	TP
DIL55-1	cocaine: procaine, 100 : 0	cocaine HCl	TP
DIL55-2	cocaine: procaine, 100 : 0	cocaine HCl	TP
DIL55-3	cocaine: procaine, 100 : 0	cocaine HCl	TP
DIL55-4	cocaine: procaine, 100 : 0	cocaine HCl	TP
DIL55-5	cocaine: procaine, 100 : 0	cocaine HCl	TP
DIL55-6	cocaine: procaine, 100 : 0	cocaine HCl	TP
DIL55-7	cocaine: procaine, 100 : 0	cocaine HCl	TP
DIL55-8	cocaine: procaine, 100 : 0	cocaine HCl	TP
DIL55-9	cocaine: procaine, 100 : 0	cocaine HCl	TP
DIL55-10	cocaine: procaine, 100 : 0	cocaine HCl	TP
DIL56-1	cocaine: inositol, 0 : 100	inositol	TN
DIL56-2	cocaine: inositol, 0 : 100	inositol	TN
DIL56-3	cocaine: inositol, 0 : 100	inositol	TN
DIL56-4	cocaine: inositol, 0 : 100	inositol	TN
DIL56-5	cocaine: inositol, 0 : 100	inositol	TN
DIL56-6	cocaine: inositol, 0 : 100	inositol	TN
DIL56-7	cocaine: inositol, 0 : 100	inositol	TN
DIL56-8	cocaine: inositol, 0 : 100	inositol	TN
DIL56-9	cocaine: inositol, 0 : 100	inconclusive	inconclusive
DIL56-10	cocaine: inositol, 0 : 100	inositol	TN
DIL57-1	cocaine: inositol, 10 : 90	cocaine HCl	TP
DIL57-2	cocaine: inositol, 10 : 90	inositol	FN
DIL57-3	cocaine: inositol, 10 : 90	inositol	FN
DIL57-4	cocaine: inositol, 10 : 90	inositol	FN
DIL57-5	cocaine: inositol, 10 : 90	inositol	FN
DIL57-6	cocaine: inositol, 10 : 90	inositol	FN
DIL57-7	cocaine: inositol, 10 : 90	inositol	FN
DIL57-8	cocaine: inositol, 10 : 90	inconclusive	inconclusive
DIL57-9	cocaine: inositol, 10 : 90	inositol	FN
DIL57-10	cocaine: inositol, 10 : 90	cocaine HCl	TP
DIL58-1	cocaine: inositol, 20 : 80	cocaine HCl	TP
DIL58-2	cocaine: inositol, 20 : 80	cocaine HCl	TP
DIL58-3	cocaine: inositol, 20 : 80	cocaine HCl	TP
DIL58-4	cocaine: inositol, 20 : 80	cocaine HCl	TP
DIL58-5	cocaine: inositol, 20 : 80	cocaine HCl	TP
DIL58-6	cocaine: inositol, 20 : 80	cocaine HCl	TP
DIL58-7	cocaine: inositol, 20 : 80	cocaine HCl	TP
DIL58-8	cocaine: inositol, 20 : 80	inconclusive	inconclusive
DIL58-9	cocaine: inositol, 20 : 80	cocaine HCl	TP
DIL58-10	cocaine: inositol, 20 : 80	cocaine HCl	TP
DIL59-1	cocaine: inositol, 30 : 70	cocaine HCl	TP
DIL59-2	cocaine: inositol, 30 : 70	cocaine HCl	TP
DIL59-3	cocaine: inositol, 30 : 70	cocaine HCl	TP
DIL59-4	cocaine: inositol, 30 : 70	cocaine HCl	TP
DIL59-5	cocaine: inositol, 30 : 70	cocaine HCl	TP
DIL59-6	cocaine: inositol, 30 : 70	cocaine HCl	TP
DIL59-7	cocaine: inositol, 30 : 70	cocaine HCl	TP
DIL59-8	cocaine: inositol, 30 : 70	cocaine HCl	TP
DIL59-9	cocaine: inositol, 30 : 70	cocaine HCl	TP

DIL15-10	cocaine: lidocaine, 30 : 70	cocaine HCl	TP
DIL16-1	cocaine: lidocaine, 40 : 60	cocaine HCl	TP
DIL16-2	cocaine: lidocaine, 40 : 60	cocaine HCl	TP
DIL16-3	cocaine: lidocaine, 40 : 60	cocaine HCl	TP
DIL16-4	cocaine: lidocaine, 40 : 60	cocaine HCl	TP
DIL16-5	cocaine: lidocaine, 40 : 60	cocaine HCl	TP
DIL16-6	cocaine: lidocaine, 40 : 60	cocaine HCl	TP
DIL16-7	cocaine: lidocaine, 40 : 60	cocaine HCl	TP
DIL16-8	cocaine: lidocaine, 40 : 60	cocaine HCl	TP
DIL16-9	cocaine: lidocaine, 40 : 60	cocaine HCl	TP
DIL16-10	cocaine: lidocaine, 40 : 60	cocaine HCl	TP
DIL17-1	cocaine: lidocaine, 50 : 50	cocaine HCl	TP
DIL17-2	cocaine: lidocaine, 50 : 50	cocaine HCl	TP
DIL17-3	cocaine: lidocaine, 50 : 50	cocaine HCl	TP
DIL17-4	cocaine: lidocaine, 50 : 50	cocaine HCl	TP
DIL17-5	cocaine: lidocaine, 50 : 50	cocaine HCl	TP
DIL17-6	cocaine: lidocaine, 50 : 50	cocaine HCl	TP
DIL17-7	cocaine: lidocaine, 50 : 50	cocaine HCl	TP
DIL17-8	cocaine: lidocaine, 50 : 50	cocaine HCl	TP
DIL17-9	cocaine: lidocaine, 50 : 50	cocaine HCl	TP
DIL17-10	cocaine: lidocaine, 50 : 50	cocaine HCl	TP
DIL18-1	cocaine: lidocaine, 60 : 40	cocaine HCl	TP
DIL18-2	cocaine: lidocaine, 60 : 40	cocaine HCl	TP
DIL18-3	cocaine: lidocaine, 60 : 40	cocaine HCl	TP
DIL18-4	cocaine: lidocaine, 60 : 40	cocaine HCl	TP
DIL18-5	cocaine: lidocaine, 60 : 40	cocaine HCl	TP
DIL18-6	cocaine: lidocaine, 60 : 40	cocaine HCl	TP
DIL18-7	cocaine: lidocaine, 60 : 40	cocaine HCl	TP
DIL18-8	cocaine: lidocaine, 60 : 40	cocaine HCl	TP
DIL18-9	cocaine: lidocaine, 60 : 40	cocaine HCl	TP
DIL18-10	cocaine: lidocaine, 60 : 40	cocaine HCl	TP
DIL19-1	cocaine: lidocaine, 70 : 30	cocaine HCl	TP
DIL19-2	cocaine: lidocaine, 70 : 30	cocaine HCl	TP
DIL19-3	cocaine: lidocaine, 70 : 30	cocaine HCl	TP
DIL19-4	cocaine: lidocaine, 70 : 30	cocaine HCl	TP
DIL19-5	cocaine: lidocaine, 70 : 30	cocaine HCl	TP
DIL19-6	cocaine: lidocaine, 70 : 30	cocaine HCl	TP
DIL19-7	cocaine: lidocaine, 70 : 30	cocaine HCl	TP
DIL19-8	cocaine: lidocaine, 70 : 30	cocaine HCl	TP
DIL19-9	cocaine: lidocaine, 70 : 30	cocaine HCl	TP
DIL19-10	cocaine: lidocaine, 70 : 30	cocaine HCl	TP
DIL20-1	cocaine: lidocaine, 80 : 20	cocaine HCl	TP
DIL20-2	cocaine: lidocaine, 80 : 20	cocaine HCl	TP
DIL20-3	cocaine: lidocaine, 80 : 20	cocaine HCl	TP
DIL20-4	cocaine: lidocaine, 80 : 20	cocaine HCl	TP
DIL20-5	cocaine: lidocaine, 80 : 20	cocaine HCl	TP
DIL20-6	cocaine: lidocaine, 80 : 20	cocaine HCl	TP
DIL20-7	cocaine: lidocaine, 80 : 20	cocaine HCl	TP
DIL20-8	cocaine: lidocaine, 80 : 20	cocaine HCl	TP
DIL20-9	cocaine: lidocaine, 80 : 20	cocaine HCl	TP
DIL20-10	cocaine: lidocaine, 80 : 20	cocaine HCl	TP
DIL21-1	cocaine: lidocaine, 90 : 10	cocaine HCl	TP
DIL21-2	cocaine: lidocaine, 90 : 10	cocaine HCl	TP
DIL21-3	cocaine: lidocaine, 90 : 10	cocaine HCl	TP
DIL21-4	cocaine: lidocaine, 90 : 10	cocaine HCl	TP
DIL21-5	cocaine: lidocaine, 90 : 10	cocaine HCl	TP
DIL21-6	cocaine: lidocaine, 90 : 10	cocaine HCl	TP
DIL21-7	cocaine: lidocaine, 90 : 10	cocaine HCl	TP
DIL21-8	cocaine: lidocaine, 90 : 10	cocaine HCl	TP
DIL21-9	cocaine: lidocaine, 90 : 10	cocaine HCl	TP
DIL21-10	cocaine: lidocaine, 90 : 10	cocaine HCl	TP
DIL22-1	cocaine: lidocaine, 100 : 0	cocaine HCl	TP
DIL22-2	cocaine: lidocaine, 100 : 0	cocaine HCl	TP
DIL22-3	cocaine: lidocaine, 100 : 0	cocaine HCl	TP
DIL22-4	cocaine: lidocaine, 100 : 0	cocaine HCl	TP
DIL22-5	cocaine: lidocaine, 100 : 0	cocaine HCl	TP
DIL22-6	cocaine: lidocaine, 100 : 0	cocaine HCl	TP
DIL22-7	cocaine: lidocaine, 100 : 0	cocaine HCl	TP
DIL22-8	cocaine: lidocaine, 100 : 0	cocaine HCl	TP
DIL22-9	cocaine: lidocaine, 100 : 0	cocaine HCl	TP
DIL22-10	cocaine: lidocaine, 100 : 0	cocaine HCl	TP
DIL23-1	cocaine: paracetamol, 0 : 100	paracetamol	TN
DIL23-2	cocaine: paracetamol, 0 : 100	paracetamol	TN
DIL23-3	cocaine: paracetamol, 0 : 100	paracetamol	TN
DIL23-4	cocaine: paracetamol, 0 : 100	paracetamol	TN
DIL23-5	cocaine: paracetamol, 0 : 100	paracetamol	TN
DIL59-10	cocaine: inositol, 30 : 70	inconclusive	inconclusive
DIL60-1	cocaine: inositol, 40 : 60	cocaine HCl	TP
DIL60-2	cocaine: inositol, 40 : 60	cocaine HCl	TP
DIL60-3	cocaine: inositol, 40 : 60	cocaine HCl	TP
DIL60-4	cocaine: inositol, 40 : 60	cocaine HCl	TP
DIL60-5	cocaine: inositol, 40 : 60	cocaine HCl	TP
DIL60-6	cocaine: inositol, 40 : 60	cocaine HCl	TP
DIL60-7	cocaine: inositol, 40 : 60	cocaine HCl	TP
DIL60-8	cocaine: inositol, 40 : 60	cocaine HCl	TP
DIL60-9	cocaine: inositol, 40 : 60	cocaine HCl	TP
DIL60-10	cocaine: inositol, 40 : 60	inconclusive	inconclusive
DIL61-1	cocaine: inositol, 50 : 50	cocaine HCl	TP
DIL61-2	cocaine: inositol, 50 : 50	cocaine HCl	TP
DIL61-3	cocaine: inositol, 50 : 50	cocaine HCl	TP
DIL61-4	cocaine: inositol, 50 : 50	cocaine HCl	TP
DIL61-5	cocaine: inositol, 50 : 50	cocaine HCl	TP
DIL61-6	cocaine: inositol, 50 : 50	cocaine HCl	TP
DIL61-7	cocaine: inositol, 50 : 50	cocaine HCl	TP
DIL61-8	cocaine: inositol, 50 : 50	cocaine HCl	TP
DIL61-9	cocaine: inositol, 50 : 50	cocaine HCl	TP
DIL61-10	cocaine: inositol, 50 : 50	inconclusive	inconclusive
DIL62-1	cocaine: inositol, 60 : 40	cocaine HCl	TP
DIL62-2	cocaine: inositol, 60 : 40	cocaine HCl	TP
DIL62-3	cocaine: inositol, 60 : 40	cocaine HCl	TP
DIL62-4	cocaine: inositol, 60 : 40	cocaine HCl	TP
DIL62-5	cocaine: inositol, 60 : 40	cocaine HCl	TP
DIL62-6	cocaine: inositol, 60 : 40	cocaine HCl	TP
DIL62-7	cocaine: inositol, 60 : 40	cocaine HCl	TP
DIL62-8	cocaine: inositol, 60 : 40	inconclusive	inconclusive
DIL62-9	cocaine: inositol, 60 : 40	cocaine HCl	TP
DIL62-10	cocaine: inositol, 60 : 40	cocaine HCl	TP
DIL63-1	cocaine: inositol, 70 : 30	cocaine HCl	TP
DIL63-2	cocaine: inositol, 70 : 30	cocaine HCl	TP
DIL63-3	cocaine: inositol, 70 : 30	cocaine HCl	TP
DIL63-4	cocaine: inositol, 70 : 30	cocaine HCl	TP
DIL63-5	cocaine: inositol, 70 : 30	cocaine HCl	TP
DIL63-6	cocaine: inositol, 70 : 30	inconclusive	inconclusive
DIL63-7	cocaine: inositol, 70 : 30	cocaine HCl	TP
DIL63-8	cocaine: inositol, 70 : 30	cocaine HCl	TP
DIL63-9	cocaine: inositol, 70 : 30	cocaine HCl	TP
DIL63-10	cocaine: inositol, 70 : 30	cocaine HCl	TP
DIL64-1	cocaine: inositol, 80 : 20	cocaine HCl	TP
DIL64-2	cocaine: inositol, 80 : 20	cocaine HCl	TP
DIL64-3	cocaine: inositol, 80 : 20	cocaine HCl	TP
DIL64-4	cocaine: inositol, 80 : 20	cocaine HCl	TP
DIL64-5	cocaine: inositol, 80 : 20	cocaine HCl	TP
DIL64-6	cocaine: inositol, 80 : 20	cocaine HCl	TP
DIL64-7	cocaine: inositol, 80 : 20	cocaine HCl	TP
DIL64-8	cocaine: inositol, 80 : 20	cocaine HCl	TP
DIL64-9	cocaine: inositol, 80 : 20	cocaine HCl	TP
DIL64-10	cocaine: inositol, 80 : 20	cocaine HCl	TP
DIL65-1	cocaine: inositol, 90 : 10	cocaine HCl	TP
DIL65-2	cocaine: inositol, 90 : 10	cocaine HCl	TP
DIL65-3	cocaine: inositol, 90 : 10	cocaine HCl	TP
DIL65-4	cocaine: inositol, 90 : 10	cocaine HCl	TP
DIL65-5	cocaine: inositol, 90 : 10	cocaine HCl	TP
DIL65-6	cocaine: inositol, 90 : 10	cocaine HCl	TP
DIL65-7	cocaine: inositol, 90 : 10	cocaine HCl	TP
DIL65-8	cocaine: inositol, 90 : 10	cocaine HCl	TP
DIL65-9	cocaine: inositol, 90 : 10	cocaine HCl	TP
DIL65-10	cocaine: inositol, 90 : 10	cocaine HCl	TP
DIL66-1	cocaine: inositol, 100 : 0	cocaine HCl	TP
DIL66-2	cocaine: inositol, 100 : 0	cocaine HCl	TP
DIL66-3	cocaine: inositol, 100 : 0	cocaine HCl	TP
DIL66-4	cocaine: inositol, 100 : 0	cocaine HCl	TP
DIL66-5	cocaine: inositol, 100 : 0	cocaine HCl	TP
DIL66-6	cocaine: inositol, 100 : 0	cocaine HCl	TP
DIL66-7	cocaine: inositol, 100 : 0	cocaine HCl	TP
DIL66-8	cocaine: inositol, 100 : 0	cocaine HCl	TP
DIL66-9	cocaine: inositol, 100 : 0	cocaine HCl	TP
DIL66-10	cocaine: inositol, 100 : 0	cocaine HCl	TP
DIL67-1	cocaine: mannitol, 0 : 100	mannitol	TN
DIL67-2	cocaine: mannitol, 0 : 100	mannitol	TN
DIL67-3	cocaine: mannitol, 0 : 100	mannitol	TN
DIL67-4	cocaine: mannitol, 0 : 100	mannitol	TN
DIL67-5	cocaine: mannitol, 0 : 100	mannitol	TN

DIL23-6	cocaine: paracetamol, 0 : 100	paracetamol	TN
DIL23-7	cocaine: paracetamol, 0 : 100	paracetamol	TN
DIL23-8	cocaine: paracetamol, 0 : 100	paracetamol	TN
DIL23-9	cocaine: paracetamol, 0 : 100	paracetamol	TN
DIL23-10	cocaine: paracetamol, 0 : 100	paracetamol	TN
DIL24-1	cocaine: paracetamol, 10 : 90	paracetamol	FN
DIL24-2	cocaine: paracetamol, 10 : 90	cocaine HCl	TP
DIL24-3	cocaine: paracetamol, 10 : 90	paracetamol	FN
DIL24-4	cocaine: paracetamol, 10 : 90	paracetamol	FN
DIL24-5	cocaine: paracetamol, 10 : 90	paracetamol	FN
DIL24-6	cocaine: paracetamol, 10 : 90	paracetamol	FN
DIL24-7	cocaine: paracetamol, 10 : 90	paracetamol	FN
DIL24-8	cocaine: paracetamol, 10 : 90	paracetamol	FN
DIL24-9	cocaine: paracetamol, 10 : 90	paracetamol	FN
DIL24-10	cocaine: paracetamol, 10 : 90	paracetamol	FN
DIL25-1	cocaine: paracetamol, 20 : 80	paracetamol	FN
DIL25-2	cocaine: paracetamol, 20 : 80	paracetamol	FN
DIL25-3	cocaine: paracetamol, 20 : 80	paracetamol	FN
DIL25-4	cocaine: paracetamol, 20 : 80	paracetamol	FN
DIL25-5	cocaine: paracetamol, 20 : 80	cocaine HCl	TP
DIL25-6	cocaine: paracetamol, 20 : 80	paracetamol	FN
DIL25-7	cocaine: paracetamol, 20 : 80	paracetamol	FN
DIL25-8	cocaine: paracetamol, 20 : 80	paracetamol	FN
DIL25-9	cocaine: paracetamol, 20 : 80	paracetamol	FN
DIL25-10	cocaine: paracetamol, 20 : 80	paracetamol	FN
DIL26-1	cocaine: paracetamol, 30 : 70	cocaine HCl	TP
DIL26-2	cocaine: paracetamol, 30 : 70	paracetamol	FN
DIL26-3	cocaine: paracetamol, 30 : 70	cocaine HCl	TP
DIL26-4	cocaine: paracetamol, 30 : 70	cocaine HCl	TP
DIL26-5	cocaine: paracetamol, 30 : 70	paracetamol	FN
DIL26-6	cocaine: paracetamol, 30 : 70	cocaine HCl	TP
DIL26-7	cocaine: paracetamol, 30 : 70	cocaine HCl	TP
DIL26-8	cocaine: paracetamol, 30 : 70	paracetamol	FN
DIL26-9	cocaine: paracetamol, 30 : 70	paracetamol	FN
DIL26-10	cocaine: paracetamol, 30 : 70	cocaine HCl	TP
DIL27-1	cocaine: paracetamol, 40 : 60	cocaine HCl	TP
DIL27-2	cocaine: paracetamol, 40 : 60	paracetamol	FN
DIL27-3	cocaine: paracetamol, 40 : 60	paracetamol	FN
DIL27-4	cocaine: paracetamol, 40 : 60	paracetamol	FN
DIL27-5	cocaine: paracetamol, 40 : 60	paracetamol	FN
DIL27-6	cocaine: paracetamol, 40 : 60	paracetamol	FN
DIL27-7	cocaine: paracetamol, 40 : 60	paracetamol	FN
DIL27-8	cocaine: paracetamol, 40 : 60	paracetamol	FN
DIL27-9	cocaine: paracetamol, 40 : 60	paracetamol	FN
DIL27-10	cocaine: paracetamol, 40 : 60	paracetamol	FN
DIL28-1	cocaine: paracetamol, 50 : 50	cocaine HCl	TP
DIL28-2	cocaine: paracetamol, 50 : 50	cocaine HCl	TP
DIL28-3	cocaine: paracetamol, 50 : 50	cocaine HCl	TP
DIL28-4	cocaine: paracetamol, 50 : 50	cocaine HCl	TP
DIL28-5	cocaine: paracetamol, 50 : 50	paracetamol	FN
DIL28-6	cocaine: paracetamol, 50 : 50	cocaine HCl	TP
DIL28-7	cocaine: paracetamol, 50 : 50	cocaine HCl	TP
DIL28-8	cocaine: paracetamol, 50 : 50	cocaine HCl	TP
DIL28-9	cocaine: paracetamol, 50 : 50	cocaine HCl	TP
DIL28-10	cocaine: paracetamol, 50 : 50	paracetamol	FN
DIL29-1	cocaine: paracetamol, 60 : 40	cocaine HCl	TP
DIL29-2	cocaine: paracetamol, 60 : 40	cocaine HCl	TP
DIL29-3	cocaine: paracetamol, 60 : 40	cocaine HCl	TP
DIL29-4	cocaine: paracetamol, 60 : 40	cocaine HCl	TP
DIL29-5	cocaine: paracetamol, 60 : 40	cocaine HCl	TP
DIL29-6	cocaine: paracetamol, 60 : 40	cocaine HCl	TP
DIL29-7	cocaine: paracetamol, 60 : 40	cocaine HCl	TP
DIL29-8	cocaine: paracetamol, 60 : 40	paracetamol	FN
DIL29-9	cocaine: paracetamol, 60 : 40	cocaine HCl	TP
DIL29-10	cocaine: paracetamol, 60 : 40	cocaine HCl	TP
DIL30-1	cocaine: paracetamol, 70 : 30	cocaine HCl	TP
DIL30-2	cocaine: paracetamol, 70 : 30	cocaine HCl	TP
DIL30-3	cocaine: paracetamol, 70 : 30	cocaine HCl	TP
DIL30-4	cocaine: paracetamol, 70 : 30	cocaine HCl	TP
DIL30-5	cocaine: paracetamol, 70 : 30	cocaine HCl	TP
DIL30-6	cocaine: paracetamol, 70 : 30	cocaine HCl	TP
DIL30-7	cocaine: paracetamol, 70 : 30	cocaine HCl	TP
DIL30-8	cocaine: paracetamol, 70 : 30	cocaine HCl	TP
DIL30-9	cocaine: paracetamol, 70 : 30	cocaine HCl	TP
DIL30-10	cocaine: paracetamol, 70 : 30	cocaine HCl	TP
DIL31-1	cocaine: paracetamol, 80 : 20	cocaine HCl	TP

DIL67-6	cocaine: mannitol, 0 : 100	inconclusive	inconclusive
DIL67-7	cocaine: mannitol, 0 : 100	mannitol	TN
DIL67-8	cocaine: mannitol, 0 : 100	mannitol	TN
DIL67-9	cocaine: mannitol, 0 : 100	inconclusive	inconclusive
DIL67-10	cocaine: mannitol, 0 : 100	mannitol	TN
DIL68-1	cocaine: mannitol, 10 : 90	cocaine HCl	TP
DIL68-2	cocaine: mannitol, 10 : 90	cocaine HCl	TP
DIL68-3	cocaine: mannitol, 10 : 90	mannitol	FN
DIL68-4	cocaine: mannitol, 10 : 90	cocaine HCl	TP
DIL68-5	cocaine: mannitol, 10 : 90	mannitol	FN
DIL68-6	cocaine: mannitol, 10 : 90	cocaine HCl	TP
DIL68-7	cocaine: mannitol, 10 : 90	cocaine HCl	TP
DIL68-8	cocaine: mannitol, 10 : 90	inconclusive	inconclusive
DIL68-9	cocaine: mannitol, 10 : 90	inconclusive	inconclusive
DIL68-10	cocaine: mannitol, 10 : 90	cocaine HCl	TP
DIL69-1	cocaine: mannitol, 20 : 80	mannitol	FN
DIL69-2	cocaine: mannitol, 20 : 80	cocaine HCl	TP
DIL69-3	cocaine: mannitol, 20 : 80	cocaine HCl	TP
DIL69-4	cocaine: mannitol, 20 : 80	cocaine HCl	TP
DIL69-5	cocaine: mannitol, 20 : 80	cocaine HCl	TP
DIL69-6	cocaine: mannitol, 20 : 80	mannitol	FN
DIL69-7	cocaine: mannitol, 20 : 80	cocaine HCl	TP
DIL69-8	cocaine: mannitol, 20 : 80	inconclusive	inconclusive
DIL69-9	cocaine: mannitol, 20 : 80	inconclusive	inconclusive
DIL69-10	cocaine: mannitol, 20 : 80	inconclusive	inconclusive
DIL70-1	cocaine: mannitol, 30 : 70	cocaine HCl	TP
DIL70-2	cocaine: mannitol, 30 : 70	mannitol	FN
DIL70-3	cocaine: mannitol, 30 : 70	cocaine HCl	TP
DIL70-4	cocaine: mannitol, 30 : 70	cocaine HCl	TP
DIL70-5	cocaine: mannitol, 30 : 70	cocaine HCl	TP
DIL70-6	cocaine: mannitol, 30 : 70	cocaine HCl	TP
DIL70-7	cocaine: mannitol, 30 : 70	mannitol	FN
DIL70-8	cocaine: mannitol, 30 : 70	inconclusive	inconclusive
DIL70-9	cocaine: mannitol, 30 : 70	cocaine HCl	TP
DIL70-10	cocaine: mannitol, 30 : 70	cocaine HCl	TP
DIL71-1	cocaine: mannitol, 40 : 60	cocaine HCl	TP
DIL71-2	cocaine: mannitol, 40 : 60	cocaine HCl	TP
DIL71-3	cocaine: mannitol, 40 : 60	cocaine HCl	TP
DIL71-4	cocaine: mannitol, 40 : 60	cocaine HCl	TP
DIL71-5	cocaine: mannitol, 40 : 60	cocaine HCl	TP
DIL71-6	cocaine: mannitol, 40 : 60	cocaine HCl	TP
DIL71-7	cocaine: mannitol, 40 : 60	cocaine HCl	TP
DIL71-8	cocaine: mannitol, 40 : 60	cocaine HCl	TP
DIL71-9	cocaine: mannitol, 40 : 60	inconclusive	inconclusive
DIL71-10	cocaine: mannitol, 40 : 60	cocaine HCl	TP
DIL72-1	cocaine: mannitol, 50 : 50	cocaine HCl	TP
DIL72-2	cocaine: mannitol, 50 : 50	cocaine HCl	TP
DIL72-3	cocaine: mannitol, 50 : 50	cocaine HCl	TP
DIL72-4	cocaine: mannitol, 50 : 50	cocaine HCl	TP
DIL72-5	cocaine: mannitol, 50 : 50	cocaine HCl	TP
DIL72-6	cocaine: mannitol, 50 : 50	mannitol	FN
DIL72-7	cocaine: mannitol, 50 : 50	cocaine HCl	TP
DIL72-8	cocaine: mannitol, 50 : 50	cocaine HCl	TP
DIL72-9	cocaine: mannitol, 50 : 50	cocaine HCl	TP
DIL72-10	cocaine: mannitol, 50 : 50	cocaine HCl	TP
DIL73-1	cocaine: mannitol, 60 : 40	cocaine HCl	TP
DIL73-2	cocaine: mannitol, 60 : 40	cocaine HCl	TP
DIL73-3	cocaine: mannitol, 60 : 40	cocaine HCl	TP
DIL73-4	cocaine: mannitol, 60 : 40	cocaine HCl	TP
DIL73-5	cocaine: mannitol, 60 : 40	cocaine HCl	TP
DIL73-6	cocaine: mannitol, 60 : 40	cocaine HCl	TP
DIL73-7	cocaine: mannitol, 60 : 40	cocaine HCl	TP
DIL73-8	cocaine: mannitol, 60 : 40	cocaine HCl	TP
DIL73-9	cocaine: mannitol, 60 : 40	inconclusive	inconclusive
DIL73-10	cocaine: mannitol, 60 : 40	cocaine HCl	TP
DIL74-1	cocaine: mannitol, 70 : 30	cocaine HCl	TP
DIL74-2	cocaine: mannitol, 70 : 30	cocaine HCl	TP
DIL74-3	cocaine: mannitol, 70 : 30	inconclusive	inconclusive
DIL74-4	cocaine: mannitol, 70 : 30	cocaine HCl	TP
DIL74-5	cocaine: mannitol, 70 : 30	cocaine HCl	TP
DIL74-6	cocaine: mannitol, 70 : 30	cocaine HCl	TP
DIL74-7	cocaine: mannitol, 70 : 30	cocaine HCl	TP
DIL74-8	cocaine: mannitol, 70 : 30	cocaine HCl	TP
DIL74-9	cocaine: mannitol, 70 : 30	cocaine HCl	TP
DIL74-10	cocaine: mannitol, 70 : 30	cocaine HCl	TP
DIL75-1	cocaine: mannitol, 80 : 20	cocaine HCl	TP

DIL31-2	cocaine: paracetamol, 80 : 20	cocaine HCl	TP
DIL31-3	cocaine: paracetamol, 80 : 20	cocaine HCl	TP
DIL31-4	cocaine: paracetamol, 80 : 20	cocaine HCl	TP
DIL31-5	cocaine: paracetamol, 80 : 20	cocaine HCl	TP
DIL31-6	cocaine: paracetamol, 80 : 20	cocaine HCl	TP
DIL31-7	cocaine: paracetamol, 80 : 20	cocaine HCl	TP
DIL31-8	cocaine: paracetamol, 80 : 20	cocaine HCl	TP
DIL31-9	cocaine: paracetamol, 80 : 20	cocaine HCl	TP
DIL31-10	cocaine: paracetamol, 80 : 20	cocaine HCl	TP
DIL32-1	cocaine: paracetamol, 90 : 10	cocaine HCl	TP
DIL32-2	cocaine: paracetamol, 90 : 10	cocaine HCl	TP
DIL32-3	cocaine: paracetamol, 90 : 10	cocaine HCl	TP
DIL32-4	cocaine: paracetamol, 90 : 10	cocaine HCl	TP
DIL32-5	cocaine: paracetamol, 90 : 10	cocaine HCl	TP
DIL32-6	cocaine: paracetamol, 90 : 10	cocaine HCl	TP
DIL32-7	cocaine: paracetamol, 90 : 10	cocaine HCl	TP
DIL32-8	cocaine: paracetamol, 90 : 10	cocaine HCl	TP
DIL32-9	cocaine: paracetamol, 90 : 10	cocaine HCl	TP
DIL32-10	cocaine: paracetamol, 90 : 10	cocaine HCl	TP
DIL33-1	cocaine: paracetamol, 100 : 0	cocaine HCl	TP
DIL33-2	cocaine: paracetamol, 100 : 0	cocaine HCl	TP
DIL33-3	cocaine: paracetamol, 100 : 0	cocaine HCl	TP
DIL33-4	cocaine: paracetamol, 100 : 0	cocaine HCl	TP
DIL33-5	cocaine: paracetamol, 100 : 0	cocaine HCl	TP
DIL33-6	cocaine: paracetamol, 100 : 0	cocaine HCl	TP
DIL33-7	cocaine: paracetamol, 100 : 0	cocaine HCl	TP
DIL33-8	cocaine: paracetamol, 100 : 0	cocaine HCl	TP
DIL33-9	cocaine: paracetamol, 100 : 0	cocaine HCl	TP
DIL33-10	cocaine: paracetamol, 100 : 0	cocaine HCl	TP
DIL34-1	cocaine: phenacetine, 0 : 100	phenacetin	TN
DIL34-2	cocaine: phenacetine, 0 : 100	phenacetin	TN
DIL34-3	cocaine: phenacetine, 0 : 100	phenacetin	TN
DIL34-4	cocaine: phenacetine, 0 : 100	phenacetin	TN
DIL34-5	cocaine: phenacetine, 0 : 100	phenacetin	TN
DIL34-6	cocaine: phenacetine, 0 : 100	phenacetin	TN
DIL34-7	cocaine: phenacetine, 0 : 100	phenacetin	TN
DIL34-8	cocaine: phenacetine, 0 : 100	phenacetin	TN
DIL34-9	cocaine: phenacetine, 0 : 100	phenacetin	TN
DIL34-10	cocaine: phenacetine, 0 : 100	phenacetin	TN
DIL35-1	cocaine: phenacetine, 10 : 90	phenacetin	FN
DIL35-2	cocaine: phenacetine, 10 : 90	phenacetin	FN
DIL35-3	cocaine: phenacetine, 10 : 90	phenacetin	FN
DIL35-4	cocaine: phenacetine, 10 : 90	cocaine HCl	TP
DIL35-5	cocaine: phenacetine, 10 : 90	phenacetin	FN
DIL35-6	cocaine: phenacetine, 10 : 90	phenacetin	FN
DIL35-7	cocaine: phenacetine, 10 : 90	phenacetin	FN
DIL35-8	cocaine: phenacetine, 10 : 90	phenacetin	FN
DIL35-9	cocaine: phenacetine, 10 : 90	phenacetin	FN
DIL35-10	cocaine: phenacetine, 10 : 90	phenacetin	FN
DIL36-1	cocaine: phenacetine, 20 : 80	phenacetin	FN
DIL36-2	cocaine: phenacetine, 20 : 80	phenacetin	FN
DIL36-3	cocaine: phenacetine, 20 : 80	phenacetin	FN
DIL36-4	cocaine: phenacetine, 20 : 80	cocaine HCl	TP
DIL36-5	cocaine: phenacetine, 20 : 80	phenacetin	FN
DIL36-6	cocaine: phenacetine, 20 : 80	cocaine HCl	TP
DIL36-7	cocaine: phenacetine, 20 : 80	cocaine HCl	TP
DIL36-8	cocaine: phenacetine, 20 : 80	phenacetin	FN
DIL36-9	cocaine: phenacetine, 20 : 80	phenacetin	FN
DIL36-10	cocaine: phenacetine, 20 : 80	phenacetin	FN
DIL37-1	cocaine: phenacetine, 30 : 70	cocaine HCl	TP
DIL37-2	cocaine: phenacetine, 30 : 70	cocaine HCl	TP
DIL37-3	cocaine: phenacetine, 30 : 70	cocaine HCl	TP
DIL37-4	cocaine: phenacetine, 30 : 70	cocaine HCl	TP
DIL37-5	cocaine: phenacetine, 30 : 70	phenacetin	FN
DIL37-6	cocaine: phenacetine, 30 : 70	cocaine HCl	TP
DIL37-7	cocaine: phenacetine, 30 : 70	cocaine HCl	TP
DIL37-8	cocaine: phenacetine, 30 : 70	cocaine HCl	TP
DIL37-9	cocaine: phenacetine, 30 : 70	cocaine HCl	TP
DIL37-10	cocaine: phenacetine, 30 : 70	cocaine HCl	TP
DIL38-1	cocaine: phenacetine, 40 : 60	cocaine HCl	TP
DIL38-2	cocaine: phenacetine, 40 : 60	cocaine HCl	TP
DIL38-3	cocaine: phenacetine, 40 : 60	cocaine HCl	TP
DIL38-4	cocaine: phenacetine, 40 : 60	cocaine HCl	TP
DIL38-5	cocaine: phenacetine, 40 : 60	phenacetin	FN
DIL38-6	cocaine: phenacetine, 40 : 60	cocaine HCl	TP
DIL38-7	cocaine: phenacetine, 40 : 60	cocaine HCl	TP
DIL75-2	cocaine: mannitol, 80 : 20	cocaine HCl	TP
DIL75-3	cocaine: mannitol, 80 : 20	cocaine HCl	TP
DIL75-4	cocaine: mannitol, 80 : 20	cocaine HCl	TP
DIL75-5	cocaine: mannitol, 80 : 20	cocaine HCl	TP
DIL75-6	cocaine: mannitol, 80 : 20	cocaine HCl	TP
DIL75-7	cocaine: mannitol, 80 : 20	cocaine HCl	TP
DIL75-8	cocaine: mannitol, 80 : 20	cocaine HCl	TP
DIL75-9	cocaine: mannitol, 80 : 20	cocaine HCl	TP
DIL75-10	cocaine: mannitol, 80 : 20	cocaine HCl	TP
DIL76-1	cocaine: mannitol, 90 : 10	cocaine HCl	TP
DIL76-2	cocaine: mannitol, 90 : 10	cocaine HCl	TP
DIL76-3	cocaine: mannitol, 90 : 10	cocaine HCl	TP
DIL76-4	cocaine: mannitol, 90 : 10	cocaine HCl	TP
DIL76-5	cocaine: mannitol, 90 : 10	cocaine HCl	TP
DIL76-6	cocaine: mannitol, 90 : 10	cocaine HCl	TP
DIL76-7	cocaine: mannitol, 90 : 10	cocaine HCl	TP
DIL76-8	cocaine: mannitol, 90 : 10	cocaine HCl	TP
DIL76-9	cocaine: mannitol, 90 : 10	cocaine HCl	TP
DIL76-10	cocaine: mannitol, 90 : 10	cocaine HCl	TP
DIL77-1	cocaine: mannitol, 100 : 0	cocaine HCl	TP
DIL77-2	cocaine: mannitol, 100 : 0	cocaine HCl	TP
DIL77-3	cocaine: mannitol, 100 : 0	cocaine HCl	TP
DIL77-4	cocaine: mannitol, 100 : 0	cocaine HCl	TP
DIL77-5	cocaine: mannitol, 100 : 0	cocaine HCl	TP
DIL77-6	cocaine: mannitol, 100 : 0	cocaine HCl	TP
DIL77-7	cocaine: mannitol, 100 : 0	cocaine HCl	TP
DIL77-8	cocaine: mannitol, 100 : 0	cocaine HCl	TP
DIL77-9	cocaine: mannitol, 100 : 0	cocaine HCl	TP
DIL77-10	cocaine: mannitol, 100 : 0	cocaine HCl	TP
DIL78-1	cocaine: levamisole, 0 : 100	levamisole	TN
DIL78-2	cocaine: levamisole, 0 : 100	levamisole	TN
DIL78-3	cocaine: levamisole, 0 : 100	levamisole	TN
DIL78-4	cocaine: levamisole, 0 : 100	levamisole	TN
DIL78-5	cocaine: levamisole, 0 : 100	levamisole	TN
DIL78-6	cocaine: levamisole, 0 : 100	levamisole	TN
DIL78-7	cocaine: levamisole, 0 : 100	levamisole	TN
DIL78-8	cocaine: levamisole, 0 : 100	levamisole	TN
DIL78-9	cocaine: levamisole, 0 : 100	levamisole	TN
DIL78-10	cocaine: levamisole, 0 : 100	levamisole	TN
DIL79-1	cocaine: levamisole, 10 : 90	levamisole	FN
DIL79-2	cocaine: levamisole, 10 : 90	cocaine HCl	TP
DIL79-3	cocaine: levamisole, 10 : 90	levamisole	FN
DIL79-4	cocaine: levamisole, 10 : 90	cocaine HCl	TP
DIL79-5	cocaine: levamisole, 10 : 90	levamisole	FN
DIL79-6	cocaine: levamisole, 10 : 90	levamisole	FN
DIL79-7	cocaine: levamisole, 10 : 90	levamisole	FN
DIL79-8	cocaine: levamisole, 10 : 90	levamisole	FN
DIL79-9	cocaine: levamisole, 10 : 90	levamisole	FN
DIL79-10	cocaine: levamisole, 10 : 90	levamisole	FN
DIL80-1	cocaine: levamisole, 20 : 80	levamisole	FN
DIL80-2	cocaine: levamisole, 20 : 80	levamisole	FN
DIL80-3	cocaine: levamisole, 20 : 80	levamisole	FN
DIL80-4	cocaine: levamisole, 20 : 80	cocaine HCl	TP
DIL80-5	cocaine: levamisole, 20 : 80	cocaine HCl	TP
DIL80-6	cocaine: levamisole, 20 : 80	levamisole	FN
DIL80-7	cocaine: levamisole, 20 : 80	levamisole	FN
DIL80-8	cocaine: levamisole, 20 : 80	cocaine HCl	TP
DIL80-9	cocaine: levamisole, 20 : 80	levamisole	FN
DIL80-10	cocaine: levamisole, 20 : 80	levamisole	FN
DIL81-1	cocaine: levamisole, 30 : 70	cocaine HCl	TP
DIL81-2	cocaine: levamisole, 30 : 70	cocaine HCl	TP
DIL81-3	cocaine: levamisole, 30 : 70	levamisole	FN
DIL81-4	cocaine: levamisole, 30 : 70	levamisole	FN
DIL81-5	cocaine: levamisole, 30 : 70	levamisole	FN
DIL81-6	cocaine: levamisole, 30 : 70	cocaine HCl	TP
DIL81-7	cocaine: levamisole, 30 : 70	cocaine HCl	TP
DIL81-8	cocaine: levamisole, 30 : 70	cocaine HCl	TP
DIL81-9	cocaine: levamisole, 30 : 70	cocaine HCl	TP
DIL81-10	cocaine: levamisole, 30 : 70	levamisole	FN
DIL82-1	cocaine: levamisole, 40 : 60	cocaine HCl	TP
DIL82-2	cocaine: levamisole, 40 : 60	cocaine HCl	TP
DIL82-3	cocaine: levamisole, 40 : 60	cocaine HCl	TP
DIL82-4	cocaine: levamisole, 40 : 60	cocaine HCl	TP
DIL82-5	cocaine: levamisole, 40 : 60	cocaine HCl	TP
DIL82-6	cocaine: levamisole, 40 : 60	levamisole	FN
DIL82-7	cocaine: levamisole, 40 : 60	cocaine HCl	TP

DIL38-8	cocaine: phenacetine, 40 : 60	cocaine HCl	TP
DIL38-9	cocaine: phenacetine, 40 : 60	phenacetin	FN
DIL38-10	cocaine: phenacetine, 40 : 60	cocaine HCl	TP
DIL39-1	cocaine: phenacetine, 50 : 50	phenacetin	FN
DIL39-2	cocaine: phenacetine, 50 : 50	phenacetin	FN
DIL39-3	cocaine: phenacetine, 50 : 50	cocaine HCl	TP
DIL39-4	cocaine: phenacetine, 50 : 50	cocaine HCl	TP
DIL39-5	cocaine: phenacetine, 50 : 50	cocaine HCl	TP
DIL39-6	cocaine: phenacetine, 50 : 50	cocaine HCl	TP
DIL39-7	cocaine: phenacetine, 50 : 50	cocaine HCl	TP
DIL39-8	cocaine: phenacetine, 50 : 50	cocaine HCl	TP
DIL39-9	cocaine: phenacetine, 50 : 50	cocaine HCl	TP
DIL39-10	cocaine: phenacetine, 50 : 50	cocaine HCl	TP
DIL40-1	cocaine: phenacetine, 60 : 40	cocaine HCl	TP
DIL40-2	cocaine: phenacetine, 60 : 40	cocaine HCl	TP
DIL40-3	cocaine: phenacetine, 60 : 40	cocaine HCl	TP
DIL40-4	cocaine: phenacetine, 60 : 40	cocaine HCl	TP
DIL40-5	cocaine: phenacetine, 60 : 40	cocaine HCl	TP
DIL40-6	cocaine: phenacetine, 60 : 40	cocaine HCl	TP
DIL40-7	cocaine: phenacetine, 60 : 40	cocaine HCl	TP
DIL40-8	cocaine: phenacetine, 60 : 40	cocaine HCl	TP
DIL40-9	cocaine: phenacetine, 60 : 40	cocaine HCl	TP
DIL40-10	cocaine: phenacetine, 60 : 40	cocaine HCl	TP
DIL41-1	cocaine: phenacetine, 70 : 30	cocaine HCl	TP
DIL41-2	cocaine: phenacetine, 70 : 30	cocaine HCl	TP
DIL41-3	cocaine: phenacetine, 70 : 30	cocaine HCl	TP
DIL41-4	cocaine: phenacetine, 70 : 30	cocaine HCl	TP
DIL41-5	cocaine: phenacetine, 70 : 30	cocaine HCl	TP
DIL41-6	cocaine: phenacetine, 70 : 30	cocaine HCl	TP
DIL41-7	cocaine: phenacetine, 70 : 30	cocaine HCl	TP
DIL41-8	cocaine: phenacetine, 70 : 30	cocaine HCl	TP
DIL41-9	cocaine: phenacetine, 70 : 30	cocaine HCl	TP
DIL41-10	cocaine: phenacetine, 70 : 30	cocaine HCl	TP
DIL42-1	cocaine: phenacetine, 80 : 20	cocaine HCl	TP
DIL42-2	cocaine: phenacetine, 80 : 20	cocaine HCl	TP
DIL42-3	cocaine: phenacetine, 80 : 20	cocaine HCl	TP
DIL42-4	cocaine: phenacetine, 80 : 20	cocaine HCl	TP
DIL42-5	cocaine: phenacetine, 80 : 20	cocaine HCl	TP
DIL42-6	cocaine: phenacetine, 80 : 20	cocaine HCl	TP
DIL42-7	cocaine: phenacetine, 80 : 20	cocaine HCl	TP
DIL42-8	cocaine: phenacetine, 80 : 20	cocaine HCl	TP
DIL42-9	cocaine: phenacetine, 80 : 20	cocaine HCl	TP
DIL42-10	cocaine: phenacetine, 80 : 20	cocaine HCl	TP
DIL43-1	cocaine: phenacetine, 90 : 10	cocaine HCl	TP
DIL43-2	cocaine: phenacetine, 90 : 10	cocaine HCl	TP
DIL43-3	cocaine: phenacetine, 90 : 10	cocaine HCl	TP
DIL43-4	cocaine: phenacetine, 90 : 10	cocaine HCl	TP
DIL43-5	cocaine: phenacetine, 90 : 10	cocaine HCl	TP
DIL43-6	cocaine: phenacetine, 90 : 10	cocaine HCl	TP
DIL43-7	cocaine: phenacetine, 90 : 10	cocaine HCl	TP
DIL43-8	cocaine: phenacetine, 90 : 10	cocaine HCl	TP
DIL43-9	cocaine: phenacetine, 90 : 10	cocaine HCl	TP
DIL43-10	cocaine: phenacetine, 90 : 10	cocaine HCl	TP
DIL44-1	cocaine: phenacetine, 100 : 0	cocaine HCl	TP
DIL44-2	cocaine: phenacetine, 100 : 0	cocaine HCl	TP
DIL44-3	cocaine: phenacetine, 100 : 0	cocaine HCl	TP
DIL44-4	cocaine: phenacetine, 100 : 0	cocaine HCl	TP
DIL44-5	cocaine: phenacetine, 100 : 0	cocaine HCl	TP
DIL44-6	cocaine: phenacetine, 100 : 0	cocaine HCl	TP
DIL44-7	cocaine: phenacetine, 100 : 0	cocaine HCl	TP
DIL44-8	cocaine: phenacetine, 100 : 0	cocaine HCl	TP
DIL44-9	cocaine: phenacetine, 100 : 0	cocaine HCl	TP
DIL44-10	cocaine: phenacetine, 100 : 0	cocaine HCl	TP

DIL82-8	cocaine: levamisole, 40 : 60	cocaine HCl	TP
DIL82-9	cocaine: levamisole, 40 : 60	cocaine HCl	TP
DIL82-10	cocaine: levamisole, 40 : 60	cocaine HCl	TP
DIL83-1	cocaine: levamisole, 50 : 50	cocaine HCl	TP
DIL83-2	cocaine: levamisole, 50 : 50	cocaine HCl	TP
DIL83-3	cocaine: levamisole, 50 : 50	cocaine HCl	TP
DIL83-4	cocaine: levamisole, 50 : 50	cocaine HCl	TP
DIL83-5	cocaine: levamisole, 50 : 50	cocaine HCl	TP
DIL83-6	cocaine: levamisole, 50 : 50	cocaine HCl	TP
DIL83-7	cocaine: levamisole, 50 : 50	cocaine HCl	TP
DIL83-8	cocaine: levamisole, 50 : 50	cocaine HCl	TP
DIL83-9	cocaine: levamisole, 50 : 50	inconclusive	inconclusive
DIL83-10	cocaine: levamisole, 50 : 50	cocaine HCl	TP
DIL84-1	cocaine: levamisole, 60 : 40	cocaine HCl	TP
DIL84-2	cocaine: levamisole, 60 : 40	cocaine HCl	TP
DIL84-3	cocaine: levamisole, 60 : 40	cocaine HCl	TP
DIL84-4	cocaine: levamisole, 60 : 40	cocaine HCl	TP
DIL84-5	cocaine: levamisole, 60 : 40	cocaine HCl	TP
DIL84-6	cocaine: levamisole, 60 : 40	cocaine HCl	TP
DIL84-7	cocaine: levamisole, 60 : 40	cocaine HCl	TP
DIL84-8	cocaine: levamisole, 60 : 40	cocaine HCl	TP
DIL84-9	cocaine: levamisole, 60 : 40	cocaine HCl	TP
DIL84-10	cocaine: levamisole, 60 : 40	cocaine HCl	TP
DIL85-1	cocaine: levamisole, 70 : 30	cocaine HCl	TP
DIL85-2	cocaine: levamisole, 70 : 30	cocaine HCl	TP
DIL85-3	cocaine: levamisole, 70 : 30	cocaine HCl	TP
DIL85-4	cocaine: levamisole, 70 : 30	cocaine HCl	TP
DIL85-5	cocaine: levamisole, 70 : 30	cocaine HCl	TP
DIL85-6	cocaine: levamisole, 70 : 30	cocaine HCl	TP
DIL85-7	cocaine: levamisole, 70 : 30	cocaine HCl	TP
DIL85-8	cocaine: levamisole, 70 : 30	cocaine HCl	TP
DIL85-9	cocaine: levamisole, 70 : 30	cocaine HCl	TP
DIL85-10	cocaine: levamisole, 70 : 30	cocaine HCl	TP
DIL86-1	cocaine: levamisole, 80 : 20	cocaine HCl	TP
DIL86-2	cocaine: levamisole, 80 : 20	cocaine HCl	TP
DIL86-3	cocaine: levamisole, 80 : 20	cocaine HCl	TP
DIL86-4	cocaine: levamisole, 80 : 20	cocaine HCl	TP
DIL86-5	cocaine: levamisole, 80 : 20	cocaine HCl	TP
DIL86-6	cocaine: levamisole, 80 : 20	cocaine HCl	TP
DIL86-7	cocaine: levamisole, 80 : 20	cocaine HCl	TP
DIL86-8	cocaine: levamisole, 80 : 20	cocaine HCl	TP
DIL86-9	cocaine: levamisole, 80 : 20	cocaine HCl	TP
DIL86-10	cocaine: levamisole, 80 : 20	cocaine HCl	TP
DIL87-1	cocaine: levamisole, 90 : 10	cocaine HCl	TP
DIL87-2	cocaine: levamisole, 90 : 10	cocaine HCl	TP
DIL87-3	cocaine: levamisole, 90 : 10	cocaine HCl	TP
DIL87-4	cocaine: levamisole, 90 : 10	cocaine HCl	TP
DIL87-5	cocaine: levamisole, 90 : 10	cocaine HCl	TP
DIL87-6	cocaine: levamisole, 90 : 10	cocaine HCl	TP
DIL87-7	cocaine: levamisole, 90 : 10	cocaine HCl	TP
DIL87-8	cocaine: levamisole, 90 : 10	cocaine HCl	TP
DIL87-9	cocaine: levamisole, 90 : 10	cocaine HCl	TP
DIL87-10	cocaine: levamisole, 90 : 10	cocaine HCl	TP
DIL88-1	cocaine: levamisole, 100 : 0	cocaine HCl	TP
DIL88-2	cocaine: levamisole, 100 : 0	cocaine HCl	TP
DIL88-3	cocaine: levamisole, 100 : 0	cocaine HCl	TP
DIL88-4	cocaine: levamisole, 100 : 0	cocaine HCl	TP
DIL88-5	cocaine: levamisole, 100 : 0	cocaine HCl	TP
DIL88-6	cocaine: levamisole, 100 : 0	cocaine HCl	TP
DIL88-7	cocaine: levamisole, 100 : 0	inconclusive	inconclusive
DIL88-8	cocaine: levamisole, 100 : 0	cocaine HCl	TP
DIL88-9	cocaine: levamisole, 100 : 0	inconclusive	inconclusive
DIL88-10	cocaine: levamisole, 100 : 0	inconclusive	inconclusive

Table S2. Results TruNarc scans for drugs-of-abuse compounds, cutting agents and non-cocaine mixtures.

scan	composition	Raman result	type
PURE1-1	cocaine HCl	cocaine HCl	TP
PURE1-2	cocaine HCl	cocaine HCl	TP
PURE1-3	cocaine HCl	cocaine HCl	TP
PURE1-4	cocaine HCl	cocaine HCl	TP
PURE1-5	cocaine HCl	cocaine HCl	TP
PURE2-1	cocaine base	cocaine base	TP
PURE2-2	cocaine base	cocaine base	TP
PURE2-3	cocaine base	cocaine base	TP
PURE2-4	cocaine base	cocaine base	TP
PURE2-5	cocaine base	cocaine base	TP
PURE3-1	GHB (liquid)	GHB	TP
PURE3-2	GHB (liquid)	GHB	TP
PURE3-3	GHB (liquid)	GHB	TP
PURE3-4	GHB (liquid)	GHB	TP
PURE3-5	GHB (liquid)	GHB	TP
PURE4-1	GHB (pure)	GHB	TP
PURE4-2	GHB (pure)	GHB	TP
PURE4-3	GHB (pure)	GHB	TP
PURE4-4	GHB (pure)	GHB	TP
PURE4-5	GHB (pure)	GHB	TP
PURE5-1	Heroin (white)	Heroin	TP
PURE5-2	Heroin (white)	Heroin	TP
PURE5-3	Heroin (white)	Heroin	TP
PURE5-4	Heroin (white)	Heroin	TP
PURE5-5	Heroin (white)	Heroin	TP
PURE6-1	Heroin (brown)	Inconclusive	inconclusive
PURE6-2	Heroin (brown)	Inconclusive	inconclusive
PURE6-3	Heroin (brown)	Inconclusive	inconclusive
PURE6-4	Heroin (brown)	Inconclusive	inconclusive
PURE6-5	Heroin (brown)	Inconclusive	inconclusive
PURE7-1	MDMA	Inconclusive	inconclusive
PURE7-2	MDMA	Inconclusive	inconclusive
PURE7-3	MDMA	MDMA	TP
PURE7-4	MDMA	MDMA	TP
PURE7-5	MDMA	Inconclusive	inconclusive
PURE8-1	Methamphetamine	Methamphetamine	TP
PURE8-2	Methamphetamine	Methamphetamine	TP
PURE8-3	Methamphetamine	Methamphetamine	TP
PURE8-4	Methamphetamine	Methamphetamine	TP
PURE8-5	Methamphetamine	Methamphetamine	TP
PURE9-1	Amphetamine	Amphetamine	TP
PURE9-2	Amphetamine	Amphetamine	TP
PURE9-3	Amphetamine	Amphetamine	TP
PURE9-4	Amphetamine	Amphetamine	TP
PURE9-5	Amphetamine	Amphetamine	TP
PURE10-1	2C-B	2C-B	TP
PURE10-2	2C-B	2C-B	TP
PURE10-3	2C-B	2C-B	TP
PURE10-4	2C-B	2C-B	TP
PURE10-5	2C-B	2C-B	TP
PURE11-1	3-CMC	3-chloromethcathinone	TN
PURE11-2	3-CMC	3-chloromethcathinone	TN
PURE11-3	3-CMC	3-chloromethcathinone	TN
PURE11-4	3-CMC	3-chloromethcathinone	TN
PURE11-5	3-CMC	3-chloromethcathinone	TN
PURE12-1	4-CMC	4-chloromethcathinone	TN
PURE12-2	4-CMC	4-chloromethcathinone	TN
PURE12-3	4-CMC	4-chloromethcathinone	TN
PURE12-4	4-CMC	4-chloromethcathinone	TN
PURE12-5	4-CMC	4-chloromethcathinone	TN
PURE13-1	6-APB	inconclusive	inconclusive
PURE13-2	6-APB	inconclusive	inconclusive
PURE13-3	6-APB	inconclusive	inconclusive
PURE13-4	6-APB	inconclusive	inconclusive
PURE13-5	6-APB	inconclusive	inconclusive
PURE14-1	2-FMA	2-fluoromethamphetamine	TN
PURE14-2	2-FMA	2-fluoromethamphetamine	TN
PURE14-3	2-FMA	2-fluoromethamphetamine	TN
PURE14-4	2-FMA	2-fluoromethamphetamine	TN
PURE14-5	2-FMA	2-fluoromethamphetamine	TN
PURE15-1	4-FA	4-fluoroamphetamine	TP
PURE15-2	4-FA	4-fluoroamphetamine	TP
PURE15-3	4-FA	4-fluoroamphetamine	TP

scan	composition	Raman result	type
NEG3-1	levamisole	levamisole	TN
NEG3-2	levamisole	levamisole	TN
NEG3-3	levamisole	levamisole	TN
NEG4-1	lidocaine	lidocaine	TN
NEG4-2	lidocaine	lidocaine	TN
NEG4-3	lidocaine	lidocaine	TN
NEG5-1	phenacetin	phenacetin	TN
NEG5-2	phenacetin	phenacetin	TN
NEG5-3	phenacetin	phenacetin	TN
NEG6-1	procaine	procaine	TN
NEG6-2	procaine	procaine	TN
NEG6-3	procaine	procaine	TN
NEG7-1	benzocaine	benzocaine	TN
NEG7-2	benzocaine	benzocaine	TN
NEG7-3	benzocaine	benzocaine	TN
NEG8-1	mannitol	mannitol	TN
NEG8-2	mannitol	mannitol	TN
NEG8-3	mannitol	mannitol	TN
NEG9-1	lactose	lactose	TN
NEG9-2	lactose	lactose	TN
NEG9-3	lactose	lactose	TN
NEG10-1	vitamin C	vitamin c	TN
NEG10-2	vitamin C	vitamin c	TN
NEG10-3	vitamin C	vitamin c	TN
NEG11-1	sugar (powdered)	confectioner's sugar	TN
NEG11-2	sugar (powdered)	confectioner's sugar	TN
NEG11-3	sugar (powdered)	confectioner's sugar	TN
NEG12-1	glucose	dextrose	TN
NEG12-2	glucose	dextrose	TN
NEG12-3	glucose	dextrose	TN
NEG13-1	boric acid	boric acid	TN
NEG13-2	boric acid	boric acid	TN
NEG13-3	boric acid	boric acid	TN
NEG14-1	diltiazem	diltiazem	TN
NEG14-2	diltiazem	diltiazem	TN
NEG14-3	diltiazem	diltiazem	TN
NEG15-1	prometazine	inconclusive	inconclusive
NEG15-2	prometazine	inconclusive	inconclusive
NEG15-3	prometazine	inconclusive	inconclusive
NEG16-1	non-dairy creamer	inconclusive	inconclusive
NEG16-2	non-dairy creamer	inconclusive	inconclusive
NEG16-3	non-dairy creamer	inconclusive	inconclusive
NEG17-1	wheat flour	corn starch	TN
NEG17-2	wheat flour	corn starch	TN
NEG17-3	wheat flour	corn starch	TN
NEG18-1	acetylsalicylic acid	aspirin	TN
NEG18-2	acetylsalicylic acid	aspirin	TN
NEG18-3	acetylsalicylic acid	aspirin	TN
NEG19-1	ketamine	ketamine	TN
NEG19-2	ketamine	ketamine	TN
NEG19-3	ketamine	ketamine	TN
NEG20-1	amphetamine	amphetamine	TP
NEG20-2	amphetamine	amphetamine	TP
NEG20-3	amphetamine	amphetamine	TP
NEG21-1	MDMA (powder)	inconclusive	inconclusive
NEG21-2	MDMA (powder)	inconclusive	inconclusive
NEG21-3	MDMA (powder)	MDMA	TP
NEG22-1	methamphetamine	methamphetamine	TP
NEG22-2	methamphetamine	methamphetamine	TP
NEG22-3	methamphetamine	methamphetamine	TP
NEG23-1	heroin (white)	heroin	TP
NEG23-2	heroin (white)	heroin	TP
NEG23-3	heroin (white)	heroin	TP
NEG24-1	sildenafil citrate tablet	inconclusive	inconclusive
NEG24-2	sildenafil citrate tablet	inconclusive	inconclusive
NEG24-3	sildenafil citrate tablet	inconclusive	inconclusive
NEG25-1	oxazepam tablet	oxazepam	TP
NEG25-2	oxazepam tablet	oxazepam	TP
NEG25-3	oxazepam tablet	oxazepam	TP
NEG26-1	temazepam tablet	clear lactose cellulose	FN
NEG26-2	temazepam tablet	flunitrazepam	FP
NEG26-3	temazepam tablet	flunitrazepam	FP
NEG27-1	mephedrone	mephedrone	TP

PURE15-4	4-FA	4-fluoroamphetamine	TP
PURE15-5	4-FA	4-fluoroamphetamine	TP
PURE16-1	ketamine	ketamine	TN
PURE16-2	ketamine	ketamine	TN
PURE16-3	ketamine	ketamine	TN
PURE16-4	ketamine	ketamine	TN
PURE16-5	ketamine	ketamine	TN
PURE17-1	3-MEC	inconclusive	inconclusive
PURE17-2	3-MEC	inconclusive	inconclusive
PURE17-3	3-MEC	inconclusive	inconclusive
PURE17-4	3-MEC	inconclusive	inconclusive
PURE17-5	3-MEC	inconclusive	inconclusive
PURE18-1	4-MEC	4-methylethcathinone	TP
PURE18-2	4-MEC	4-methylethcathinone	TP
PURE18-3	4-MEC	4-methylethcathinone	TP
PURE18-4	4-MEC	4-methylethcathinone	TP
PURE18-5	4-MEC	4-methylethcathinone	TP
PURE19-1	2-MMC	2-methylmethcathinone	TN
PURE19-2	2-MMC	2-methylmethcathinone	TN
PURE19-3	2-MMC	2-methylmethcathinone	TN
PURE19-4	2-MMC	2-methylmethcathinone	TN
PURE19-5	2-MMC	2-methylmethcathinone	TN
PURE20-1	3-MMC	3-methylmethcathinone	TN
PURE20-2	3-MMC	3-methylmethcathinone	TN
PURE20-3	3-MMC	3-methylmethcathinone	TN
PURE20-4	3-MMC	3-methylmethcathinone	TN
PURE20-5	3-MMC	3-methylmethcathinone	TN
PURE21-1	4-MMC	mephedrone	TP
PURE21-2	4-MMC	mephedrone	TP
PURE21-3	4-MMC	mephedrone	TP
PURE21-4	4-MMC	mephedrone	TP
PURE21-5	4-MMC	mephedrone	TP
NEG1-1	paracetamol	paracetamol	TN
NEG1-2	paracetamol	paracetamol	TN
NEG1-3	paracetamol	paracetamol	TN
NEG2-1	caffeine	caffeine	TN
NEG2-2	caffeine	caffeine	TN
NEG2-3	caffeine	caffeine	TN

NEG27-2	mephedrone	mephedrone	TP
NEG27-3	mephedrone	mephedrone	TP
NEG28-1	4-FA	4-FA	TP
NEG28-2	4-FA	4-FA	TP
NEG28-3	4-FA	4-FA	TP
NEG29-1	paracetamol :caffein, 1:1	paracetamol	TN
NEG29-2	paracetamol :caffein, 1:1	paracetamol	TN
NEG29-3	paracetamol :caffein, 1:1	paracetamol	TN
NEG30-1	levamisole:lidocaine, 1:1	levamisole, lidocaine	TN
NEG30-2	levamisole:lidocaine, 1:1	levamisole, lidocaine	TN
NEG30-3	levamisole:lidocaine, 1:1	levamisole, lidocaine	TN
NEG31-1	levamisole:paracetamol:lidocaine, 1:1:1	paracetamol	TN
NEG31-2	levamisole:paracetamol:lidocaine, 1:1:1	paracetamol	TN
NEG31-3	levamisole:paracetamol:lidocaine, 1:1:1	paracetamol	TN
NEG32-1	levamisole:phenacetin, 1:1	phenacetin, levamisole	TN
NEG32-2	levamisole:phenacetin, 1:1	phenacetin, levamisole	TN
NEG32-3	levamisole:phenacetin, 1:1	phenacetin, levamisole	TN
NEG33-1	phenacetin:lidocaine, 1:1	phenacetin, lidocaine	TN
NEG33-2	phenacetin:lidocaine, 1:1	phenacetin, lidocaine	TN
NEG33-3	phenacetin:lidocaine, 1:1	phenacetin, lidocaine	TN
NEG34-1	phenacetin:procaine, 1:1	procaine, phenacetin	TN
NEG34-2	phenacetin:procaine, 1:1	procaine, phenacetin	TN
NEG34-3	phenacetin:procaine, 1:1	procaine, phenacetin	TN
NEG35-1	levamisole:phenacetin:procaine, 1:1:1	levamisole, procaine,	TN
NEG35-2	levamisole:phenacetin:procaine, 1:1:1	levamisole, procaine,	TN
NEG35-3	levamisole:phenacetin:procaine, 1:1:1	levamisole, procaine,	TN
NEG36-1	paracetamol:phenacetin, 1:1	paracetamol	TN
NEG36-2	paracetamol:phenacetin, 1:1	paracetamol	TN
NEG36-3	paracetamol:phenacetin, 1:1	paracetamol	TN
NEG37-1	diazepam tablet 10 mg	diazepam	TN
NEG37-2	diazepam tablet 10 mg	diazepam	TN
NEG37-3	diazepam tablet 10 mg	diazepam	TN
NEG38-1	methylphenidate 10 mg tablet	inconclusive	inconclusive
NEG38-2	methylphenidate 10 mg tablet	inconclusive	inconclusive
NEG38-3	methylphenidate 10 mg tablet	inconclusive	inconclusive
NEG39-1	smartshop blend mix caffeine,	mannitol caffeine	TN
NEG39-2	smartshop blend mix caffeine,	mannitol caffeine	TN
NEG39-3	smartshop blend mix caffeine,	mannitol caffeine	TN

Table S3. Results TruNarc scans for cocaine-containing case samples.

scan	composition	Raman result	type
POS2-1	cocaine base (78.2%), levamisole	Cocaine base	TP
POS2-2	cocaine base (78.2%), levamisole	Cocaine base	TP
POS2-3	cocaine base (78.2%), levamisole	Cocaine base	TP
POS3-1	cocaine HCl (63.2%), levamisole	Cocaine HCl	TP
POS3-2	cocaine HCl (63.2%), levamisole	Cocaine HCl	TP
POS3-3	cocaine HCl (63.2%), levamisole	Cocaine HCl	TP
POS4-1	cocaine HCl (80%), caffein	Cocaine HCl	TP
POS4-2	cocaine HCl (80%), caffein	Cocaine HCl	TP
POS4-3	cocaine HCl (80%), caffein	Cocaine HCl	TP
POS5-1	cocaine HCl (43.8%), levamisole, inositol	Cocaine HCl	TP
POS5-2	cocaine HCl (43.8%), levamisole, inositol	Cocaine HCl	TP
POS5-3	cocaine HCl (43.8%), levamisole, inositol	Cocaine HCl	TP
POS6-1	cocaine HCl (79.6%)	Cocaine HCl	TP
POS6-2	cocaine HCl (79.6%)	Cocaine HCl	TP
POS6-3	cocaine HCl (79.6%)	Cocaine HCl	TP
POS10-1	cocaine base (55.4%), phenacetin, levamisole	Cocaine HCl	TP
POS10-2	cocaine base (55.4%), phenacetin, levamisole	Cocaine HCl	TP
POS10-3	cocaine base (55.4%), phenacetin, levamisole	Cocaine base	TP
POS11-1	cocaine HCl (45.2%), paracetamol	Cocaine HCl	TP
POS11-2	cocaine HCl (45.2%), paracetamol	Cocaine HCl	TP
POS11-3	cocaine HCl (45.2%), paracetamol	Cocaine HCl	TP
POS12-1	cocaine HCl (58.2%), glucose	Cocaine HCl	TP
POS12-2	cocaine HCl (58.2%), glucose	Cocaine HCl	TP
POS12-3	cocaine HCl (58.2%), glucose	Cocaine HCl	TP
POS13-1	cocaine base (94.2%)	Cocaine base	TP
POS13-2	cocaine base (94.2%)	Cocaine base	TP
POS13-3	cocaine base (94.2%)	Cocaine base	TP
POS14-1	cocaine base (68%), caffein, phenacetin, levamisole	Cocaine base	TP
POS14-2	cocaine base (68%), caffein, phenacetin, levamisole	Cocaine base	TP
POS14-3	cocaine base (68%), caffein, phenacetin, levamisole	Cocaine base	TP
POS15-1	cocaine HCl (81.4%), levamisole	Cocaine HCl	TP
POS15-2	cocaine HCl (81.4%), levamisole	Cocaine HCl	TP
POS15-3	cocaine HCl (81.4%), levamisole	Cocaine HCl	TP
POS17-1	cocaine HCl (39.6%), phenacetin,hydroxyzine, inositol	Cocaine HCl	TP
POS17-2	cocaine HCl (39.6%), phenacetin,hydroxyzine, inositol	Cocaine HCl	TP
POS17-3	cocaine HCl (39.6%), phenacetin,hydroxyzine, inositol	Cocaine HCl	TP
POS19-1	cocaine HCl (47.2%), caffein, phenacetin, tetracaine	Cocaine HCl	TP
POS19-2	cocaine HCl (47.2%), caffein, phenacetin, tetracaine	Cocaine HCl	TP
POS19-3	cocaine HCl (47.2%), caffein, phenacetin, tetracaine	Cocaine HCl	TP
POS20-1	cocaine HCl (80.7%)	Cocaine HCl	TP
POS20-2	cocaine HCl (80.7%)	Cocaine HCl	TP
POS20-3	cocaine HCl (80.7%)	Cocaine HCl	TP
POS21-1	cocaine HCl (81.1%)	Cocaine HCl	TP
POS21-2	cocaine HCl (81.1%)	Cocaine HCl	TP
POS21-3	cocaine HCl (81.1%)	Cocaine HCl	TP
POS22-1	cocaine HCl (74.2%), levamisole	Cocaine HCl	TP
POS22-2	cocaine HCl (74.2%), levamisole	Cocaine HCl	TP
POS22-3	cocaine HCl (74.2%), levamisole	Cocaine HCl	TP
POS24-1	cocaine HCl (80.8%)	Cocaine HCl	TP
POS24-2	cocaine HCl (80.8%)	Cocaine HCl	TP
POS24-3	cocaine HCl (80.8%)	Cocaine HCl	TP
POS26-1	cocaine base (58.5%), caffein, phenacetin, levamisole	Cocaine base	TP
POS26-2	cocaine base (58.5%), caffein, phenacetin, levamisole	Cocaine base	TP
POS26-3	cocaine base (58.5%), caffein, phenacetin, levamisole	Cocaine base	TP
POS29-1	cocaine base (99.4%)	Cocaine base	TP
POS29-2	cocaine base (99.4%)	Cocaine base	TP
POS29-3	cocaine base (99.4%)	Cocaine base	TP
POS30-1	cocaine HCl (79.1%)	Cocaine HCl	TP
POS30-2	cocaine HCl (79.1%)	Cocaine HCl	TP
POS30-3	cocaine HCl (79.1%)	Cocaine HCl	TP
POS31-1	cocaine base (62.1%), phenacetin, levamisole	Cocaine base	TP
POS31-2	cocaine base (62.1%), phenacetin, levamisole	Cocaine base	TP
POS31-3	cocaine base (62.1%), phenacetin, levamisole	Cocaine base	TP
POS33-1	cocaine HCl (68.4%), levamisole	Cocaine HCl	TP
POS33-2	cocaine HCl (68.4%), levamisole	Cocaine HCl	TP
POS33-3	cocaine HCl (68.4%), levamisole	Cocaine HCl	TP
POS34-1	cocaine base (95.9%)	Cocaine base	TP
POS34-2	cocaine base (95.9%)	Cocaine base	TP
POS34-3	cocaine base (95.9%)	Cocaine base	TP
POS35-1	cocaine HCl (29.6%), levamisole, lactose	Cocaine HCl	TP
POS35-2	cocaine HCl (29.6%), levamisole, lactose	Cocaine HCl	TP
POS35-3	cocaine HCl (29.6%), levamisole, lactose	Cocaine HCl	TP
POS36-1	cocaine base (85.9%), phenacetin	Cocaine base	TP

POS36-2	cocaine base (85.9%), phenacetin	Cocaine base	TP
POS36-3	cocaine base (85.9%), phenacetin	Cocaine base	TP
POS38-1	cocaine HCl (83.3%)	Cocaine HCl	TP
POS38-2	cocaine HCl (83.3%)	Cocaine HCl	TP
POS38-3	cocaine HCl (83.3%)	Cocaine HCl	TP
POS39-1	cocaine base (99.2%)	Cocaine base	TP
POS39-2	cocaine base (99.2%)	Cocaine base	TP
POS39-3	cocaine base (99.2%)	Cocaine base	TP
POS40-1	cocaine base (85.6%), levamisole	Cocaine base	TP
POS40-2	cocaine base (85.6%), levamisole	Cocaine base	TP
POS40-3	cocaine base (85.6%), levamisole	Cocaine base	TP
POS43-1	cocaine HCl (63.4%) mannitol	Cocaine HCl	TP
POS43-2	cocaine HCl (63.4%) mannitol	Cocaine HCl	TP
POS43-3	cocaine HCl (63.4%) mannitol	Cocaine HCl	TP
POS44-1	cocaine HCl (82.8%)	Cocaine HCl	TP
POS44-2	cocaine HCl (82.8%)	Cocaine HCl	TP
POS44-3	cocaine HCl (82.8%)	Cocaine HCl	TP
POS45-1	cocaine base (98.2%)	Cocaine base	TP
POS45-2	cocaine base (98.2%)	Cocaine base	TP
POS45-3	cocaine base (98.2%)	Cocaine base	TP
POS47-1	cocaine HCl (46.1%), levamisole, inositol, mannitol	Cocaine HCl	TP
POS47-2	cocaine HCl (46.1%), levamisole, inositol, mannitol	Cocaine HCl	TP
POS47-3	cocaine HCl (46.1%), levamisole, inositol, mannitol	Cocaine HCl	TP
POS48-1	cocaine HCl (79.6%), levamisole	Cocaine HCl	TP
POS48-2	cocaine HCl (79.6%), levamisole	Cocaine HCl	TP
POS48-3	cocaine HCl (79.6%), levamisole	Cocaine HCl	TP
POS49-1	cocaine HCl (85.6%), phenacetin	Cocaine HCl	TP
POS49-2	cocaine HCl (85.6%), phenacetin	Cocaine HCl	TP
POS49-3	cocaine HCl (85.6%), phenacetin	Cocaine HCl	TP
POS51-1	cocaine HCl (45.3%), caffein, phenacetin, mannitol	Cocaine HCl	TP
POS51-2	cocaine HCl (45.3%), caffein, phenacetin, mannitol	Cocaine HCl	TP
POS51-3	cocaine HCl (45.3%), caffein, phenacetin, mannitol	Cocaine HCl	TP
POS52-1	cocaine base (98%)	Cocaine base	TP
POS52-2	cocaine base (98%)	Cocaine base	TP
POS52-3	cocaine base (98%)	Cocaine base	TP
POS54-1	cocaine HCl (55.8%), phenacetin, levamisole	Cocaine HCl	TP
POS54-2	cocaine HCl (55.8%), phenacetin, levamisole	Cocaine HCl	TP
POS54-3	cocaine HCl (55.8%), phenacetin, levamisole	Cocaine HCl	TP
POS55-1	cocaine HCl (28.2%), caffein, phenacetin, levamisole, inositol	Cocaine HCl	TP
POS55-2	cocaine HCl (28.2%), caffein, phenacetin, levamisole, inositol	Cocaine HCl	TP
POS55-3	cocaine HCl (28.2%), caffein, phenacetin, levamisole, inositol	Cocaine HCl	TP
POS56-1	cocaine HCl (51.9%), levamisole, inositol	Cocaine HCl	TP
POS56-2	cocaine HCl (51.9%), levamisole, inositol	Cocaine HCl	TP
POS56-3	cocaine HCl (51.9%), levamisole, inositol	Cocaine HCl	TP
POS57-1	cocaine HCl (44.3%) inositol	Cocaine HCl	TP
POS57-2	cocaine HCl (44.3%) inositol	Cocaine HCl	TP
POS57-3	cocaine HCl (44.3%) inositol	Cocaine HCl	TP
POS58-1	cocaine HCl (19.1%), lidocaine, mannitol	Lidocaine, Mannitol	FN
POS58-2	cocaine HCl (19.1%), lidocaine, mannitol	Cocaine HCl	TP
POS58-3	cocaine HCl (19.1%), lidocaine, mannitol	Cocaine	TP
POS59-1	cocaine base (53.4%), caffein, phenacetin	Cocaine base	TP
POS59-2	cocaine base (53.4%), caffein, phenacetin	Cocaine base	TP
POS59-3	cocaine base (53.4%), caffein, phenacetin	Cocaine base	TP
POS60-1	cocaine base (69.2%), phenacetin	Cocaine base	TP
POS60-2	cocaine base (69.2%), phenacetin	Cocaine base	TP
POS60-3	cocaine base (69.2%), phenacetin	Cocaine base	TP
POS61-1	cocaine base (56.1%), phenacetin, levamisole	Cocaine base	TP
POS61-2	cocaine base (56.1%), phenacetin, levamisole	Cocaine base	TP
POS61-3	cocaine base (56.1%), phenacetin, levamisole	Cocaine base	TP
POS62-1	cocaine base (71.1%), phenacetin	Cocaine base	TP
POS62-2	cocaine base (71.1%), phenacetin	Cocaine base	TP
POS62-3	cocaine base (71.1%), phenacetin	Cocaine base	TP
POS63-1	cocaine HCl (79.3%)	Cocaine HCl	TP
POS63-2	cocaine HCl (79.3%)	Cocaine HCl	TP
POS63-3	cocaine HCl (79.3%)	Cocaine HCl	TP
POS66-1	cocaine HCl (76.3%) inositol	Cocaine HCl	TP
POS66-2	cocaine HCl (76.3%) inositol	Cocaine HCl	TP
POS66-3	cocaine HCl (76.3%) inositol	Cocaine HCl	TP
POS67-1	cocaine HCl (85.5%)	Cocaine HCl	TP
POS67-2	cocaine HCl (85.5%)	Cocaine HCl	TP
POS67-3	cocaine HCl (85.5%)	Cocaine HCl	TP
POS68-1	cocaine base (89.1%)	Cocaine base	TP
POS68-2	cocaine base (89.1%)	Cocaine base	TP
POS68-3	cocaine base (89.1%)	Cocaine base	TP
POS69-1	cocaine HCl (47.6%), caffein, phenacetin, mannitol	Cocaine HCl	TP
POS69-2	cocaine HCl (47.6%), caffein, phenacetin, mannitol	Cocaine HCl	TP

POS69-3	cocaine HCl (47.6%), caffein, phenacetin, mannitol	Cocaine HCl	TP
POS70-1	cocaine base (85.9%), phenacetin	Cocaine base	TP
POS70-2	cocaine base (85.9%), phenacetin	Cocaine base	TP
POS70-3	cocaine base (85.9%), phenacetin	Cocaine base	TP
POS71-1	cocaine HCl (60.3%), levamisole	Cocaine HCl	TP
POS71-2	cocaine HCl (60.3%), levamisole	Cocaine HCl	TP
POS71-3	cocaine HCl (60.3%), levamisole	Cocaine HCl	TP
POS72-1	cocaine base (85.4%)	Cocaine base	TP
POS72-2	cocaine base (85.4%)	Cocaine base	TP
POS72-3	cocaine base (85.4%)	Cocaine base	TP
POS73-1	cocaine base (80%), levamisole	Cocaine base	TP
POS73-2	cocaine base (80%), levamisole	Cocaine base	TP
POS73-3	cocaine base (80%), levamisole	Cocaine base	TP
POS74-1	cocaine HCl (66.3%), lidocaine	Cocaine HCl	TP
POS74-2	cocaine HCl (66.3%), lidocaine	Cocaine HCl	TP
POS74-3	cocaine HCl (66.3%), lidocaine	Cocaine HCl	TP
POS75-1	cocaine HCl (22.5%), caffein, levamisole, inositol, mannitol	Cocaine HCl	TP
POS75-2	cocaine HCl (22.5%), caffein, levamisole, inositol, mannitol	Cocaine HCl	TP
POS75-3	cocaine HCl (22.5%), caffein, levamisole, inositol, mannitol	Cocaine HCl	TP
POS76-1	cocaine HCl (45.4%), levamisole, inositol	Cocaine HCl	TP
POS76-2	cocaine HCl (45.4%), levamisole, inositol	Cocaine HCl	TP
POS76-3	cocaine HCl (45.4%), levamisole, inositol	Cocaine HCl	TP
POS77-1	cocaine HCl (69.4%), lidocaine, inositol, mannitol	Cocaine HCl	TP
POS77-2	cocaine HCl (69.4%), lidocaine, inositol, mannitol	Cocaine HCl	TP
POS77-3	cocaine HCl (69.4%), lidocaine, inositol, mannitol	Cocaine HCl	TP
POS80-1	cocaine base (68.8%), phenacetin	Cocaine base	TP
POS80-2	cocaine base (68.8%), phenacetin	Cocaine base	TP
POS80-3	cocaine base (68.8%), phenacetin	Cocaine base	TP
POS82-1	cocaine HCl (80.6%)	Cocaine HCl	TP
POS82-2	cocaine HCl (80.6%)	Cocaine HCl	TP
POS82-3	cocaine HCl (80.6%)	Cocaine HCl	TP
POS83-1	cocaine HCl (24.6%), levamisole	Cocaine HCl	TP
POS83-2	cocaine HCl (24.6%), levamisole	Cocaine HCl	TP
POS83-3	cocaine HCl (24.6%), levamisole	Cocaine HCl	TP
POS84-1	cocaine base (79.7%), phenacetin, levamisole	Cocaine base	TP
POS84-2	cocaine base (79.7%), phenacetin, levamisole	Cocaine base	TP
POS84-3	cocaine base (79.7%), phenacetin, levamisole	Cocaine base	TP
POS85-1	cocaine base (66.9%), levamisole	Cocaine base	TP
POS85-2	cocaine base (66.9%), levamisole	Cocaine base	TP
POS85-3	cocaine base (66.9%), levamisole	Cocaine base	TP
POS86-1	cocaine base (75.8%), phenacetin	Cocaine base	TP
POS86-2	cocaine base (75.8%), phenacetin	Cocaine base	TP
POS86-3	cocaine base (75.8%), phenacetin	Cocaine base	TP
POS87-1	cocaine base (98.1%)	Cocaine base	TP
POS87-2	cocaine base (98.1%)	Cocaine base	TP
POS87-3	cocaine base (98.1%)	Cocaine base	TP
POS88-1	cocaine HCl (64.1%), phenacetin, levamisole, inositol, mannitol	Cocaine HCl	TP
POS88-2	cocaine HCl (64.1%), phenacetin, levamisole, inositol, mannitol	Cocaine HCl	TP
POS88-3	cocaine HCl (64.1%), phenacetin, levamisole, inositol, mannitol	Cocaine HCl	TP
POS89-1	cocaine HCl (63.4%), levamisole	Cocaine HCl	TP
POS89-2	cocaine HCl (63.4%), levamisole	Cocaine HCl	TP
POS89-3	cocaine HCl (63.4%), levamisole	Cocaine HCl	TP
POS90-1	cocaine HCl (62.6%), caffein, levamisole	Cocaine HCl	TP
POS90-2	cocaine HCl (62.6%), caffein, levamisole	Cocaine HCl	TP
POS90-3	cocaine HCl (62.6%), caffein, levamisole	Cocaine HCl	TP
POS92-1	cocaine HCl (72.7%), inositol	Cocaine HCl	TP
POS92-2	cocaine HCl (72.7%), inositol	Cocaine HCl	TP
POS92-3	cocaine HCl (72.7%), inositol	Cocaine HCl	TP
POS93-1	cocaine HCl (83.9%)	Cocaine HCl	TP
POS93-2	cocaine HCl (83.9%)	Cocaine HCl	TP
POS93-3	cocaine HCl (83.9%)	Cocaine HCl	TP
POS94-1	cocaine base (51.4%), phenacetin, levamisole	Cocaine base	TP
POS94-2	cocaine base (51.4%), phenacetin, levamisole	Cocaine base	TP
POS94-3	cocaine base (51.4%), phenacetin, levamisole	Cocaine base	TP
POS95-1	cocaine HCl (80.4%)	Cocaine HCl	TP
POS95-2	cocaine HCl (80.4%)	Cocaine HCl	TP
POS95-3	cocaine HCl (80.4%)	Cocaine HCl	TP
POS99-1	cocaine base (48.8%), phenacetin	Cocaine base	TP
POS99-2	cocaine base (48.8%), phenacetin	Cocaine base	TP
POS99-3	cocaine base (48.8%), phenacetin	Cocaine base	TP
POS105-1	cocaine HCl (85.4%)	Cocaine HCl	TP
POS105-2	cocaine HCl (85.4%)	Cocaine HCl	TP
POS105-3	cocaine HCl (85.4%)	Cocaine HCl	TP
POS106-1	cocaine base (93.1%)	Cocaine base	TP
POS106-2	cocaine base (93.1%)	Cocaine base	TP
POS106-3	cocaine base (93.1%)	Cocaine base	TP

POS109-1	cocaine base (31.5%), levamisole	Inconclusive	inconclusive
POS109-2	cocaine base (31.5%), levamisole	Inconclusive	inconclusive
POS109-3	cocaine base (31.5%), levamisole	Inconclusive	inconclusive
POS109-4	cocaine base (31.5%), levamisole	Inconclusive	inconclusive
POS111-1	cocaine base (82.4%)	Cocaine base	TP
POS111-2	cocaine base (82.4%)	Cocaine base	TP
POS111-3	cocaine base (82.4%)	Cocaine base	TP
POS112-1	cocaine HCl (68.4%)	Cocaine HCl	TP
POS112-2	cocaine HCl (68.4%)	Cocaine HCl	TP
POS112-3	cocaine HCl (68.4%)	Cocaine HCl	TP
POS113-1	cocaine HCl (63.4%), levamisole, procaine	Cocaine HCl	TP
POS113-2	cocaine HCl (63.4%), levamisole, procaine	Cocaine HCl	TP
POS113-3	cocaine HCl (63.4%), levamisole, procaine	Cocaine HCl	TP
POS115-1	cocaine HCl (84%)	Cocaine HCl	TP
POS115-2	cocaine HCl (84%)	Cocaine HCl	TP
POS115-3	cocaine HCl (84%)	Cocaine HCl	TP
POS116-1	cocaine HCl (72.6%)	Cocaine HCl	TP
POS116-2	cocaine HCl (72.6%)	Cocaine HCl	TP
POS116-3	cocaine HCl (72.6%)	Cocaine HCl	TP
POS117-1	cocaine HCl (67.9%), levamisole	Cocaine HCl	TP
POS117-2	cocaine HCl (67.9%), levamisole	Cocaine HCl	TP
POS117-3	cocaine HCl (67.9%), levamisole	Cocaine HCl	TP
POS118-1	cocaine HCl (83%)	Cocaine HCl	TP
POS118-2	cocaine HCl (83%)	Cocaine HCl	TP
POS118-3	cocaine HCl (83%)	Cocaine HCl	TP
POS119-1	cocaine base (48.8%), phenacetin, levamisole	Cocaine base	TP
POS119-2	cocaine base (48.8%), phenacetin, levamisole	Cocaine base	TP
POS119-3	cocaine base (48.8%), phenacetin, levamisole	Cocaine base	TP
POS120-1	cocaine HCl (79.7%)	Cocaine HCl	TP
POS120-2	cocaine HCl (79.7%)	Cocaine HCl	TP
POS120-3	cocaine HCl (79.7%)	Cocaine HCl	TP
POS121-1	cocaine HCl (44.1%), cocaine, mannitol	Cocaine HCl	TP
POS121-2	cocaine HCl (44.1%), cocaine, mannitol	Cocaine HCl	TP
POS121-3	cocaine HCl (44.1%), cocaine, mannitol	Cocaine HCl	TP
POS122-1	cocaine HCl (46.4%), phenacetin, levamisole	Cocaine HCl	TP
POS122-2	cocaine HCl (46.4%), phenacetin, levamisole	Cocaine HCl	TP
POS122-3	cocaine HCl (46.4%), phenacetin, levamisole	Cocaine HCl	TP
POS123-1	cocaine HCl (77.9%)	Cocaine HCl	TP
POS123-2	cocaine HCl (77.9%)	Cocaine HCl	TP
POS123-3	cocaine HCl (77.9%)	Cocaine HCl	TP
POS124-1	cocaine HCl (80.8%), mannitol	Cocaine HCl	TP
POS124-2	cocaine HCl (80.8%), mannitol	Cocaine HCl	TP
POS124-3	cocaine HCl (80.8%), mannitol	Cocaine HCl	TP
POS125-1	cocaine HCl (76.8%)	Cocaine HCl	TP
POS125-2	cocaine HCl (76.8%)	Cocaine HCl	TP
POS125-3	cocaine HCl (76.8%)	Cocaine HCl	TP

Note. Cocaine percentages are given in wt% of cocaine base.

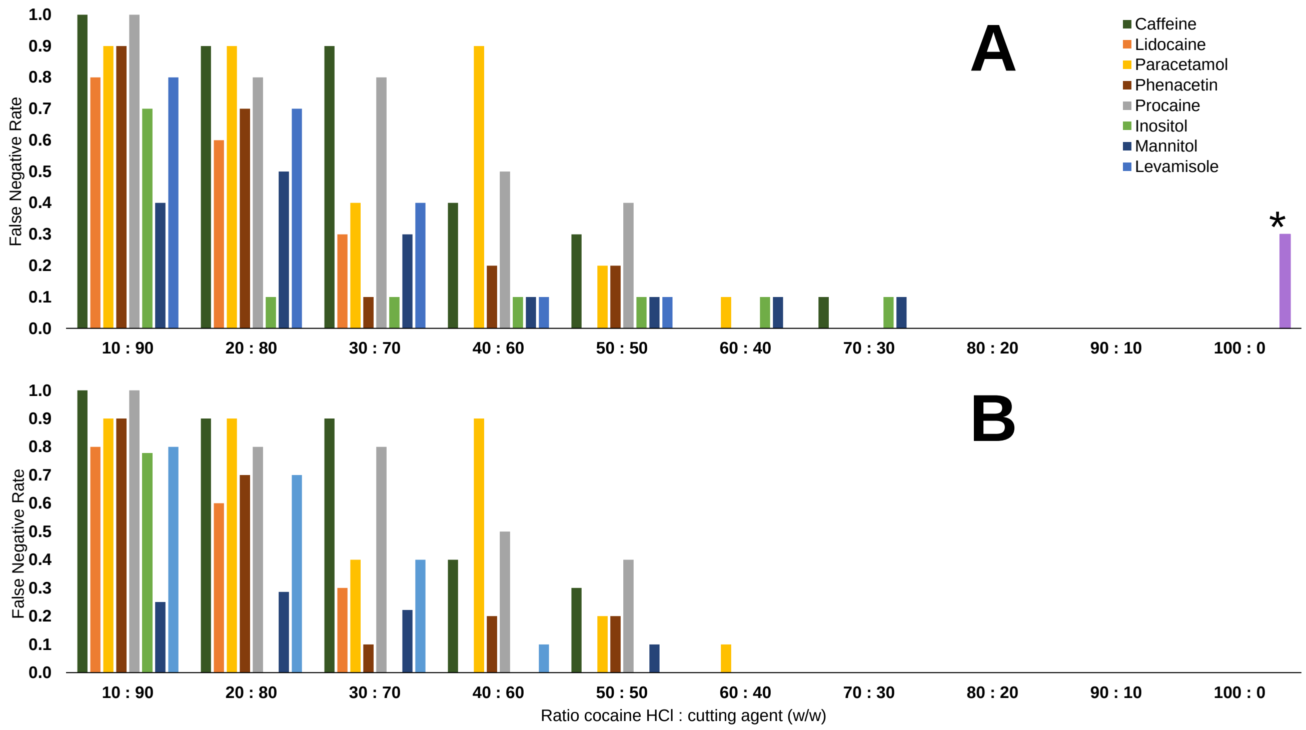

Figure S1. False-negative results of the TruNarc handheld Raman spectrometer for binary cocaine mixtures with 8 common cutting-agents at concentrations ranging from 10 – 100 wt% cocaine. 10-fold replicate scans per sample; no special instructions for analyzing. False-negative rates with (A) and without (B) inconclusive scan results. (*) indicates inconclusive results for pure cocaine (3 out of 80 scans).

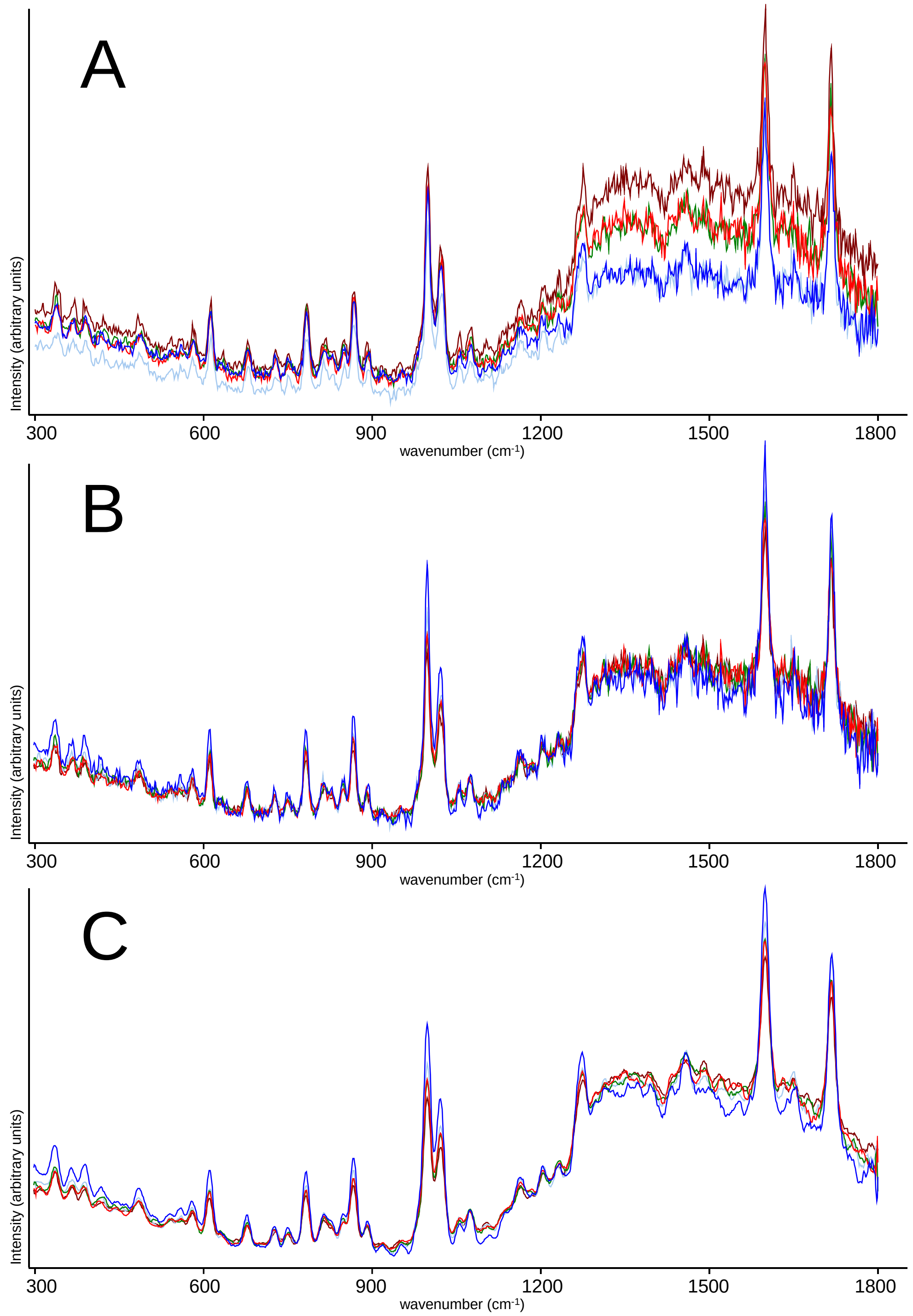

Figure S2. Raman spectra of 5 replicate scans of a single cocaine HCl sample. Raw spectral data (A), spectral data after SNV preprocessing (B) and SVN with subsequent 9 datapoint SG smoothing (C).

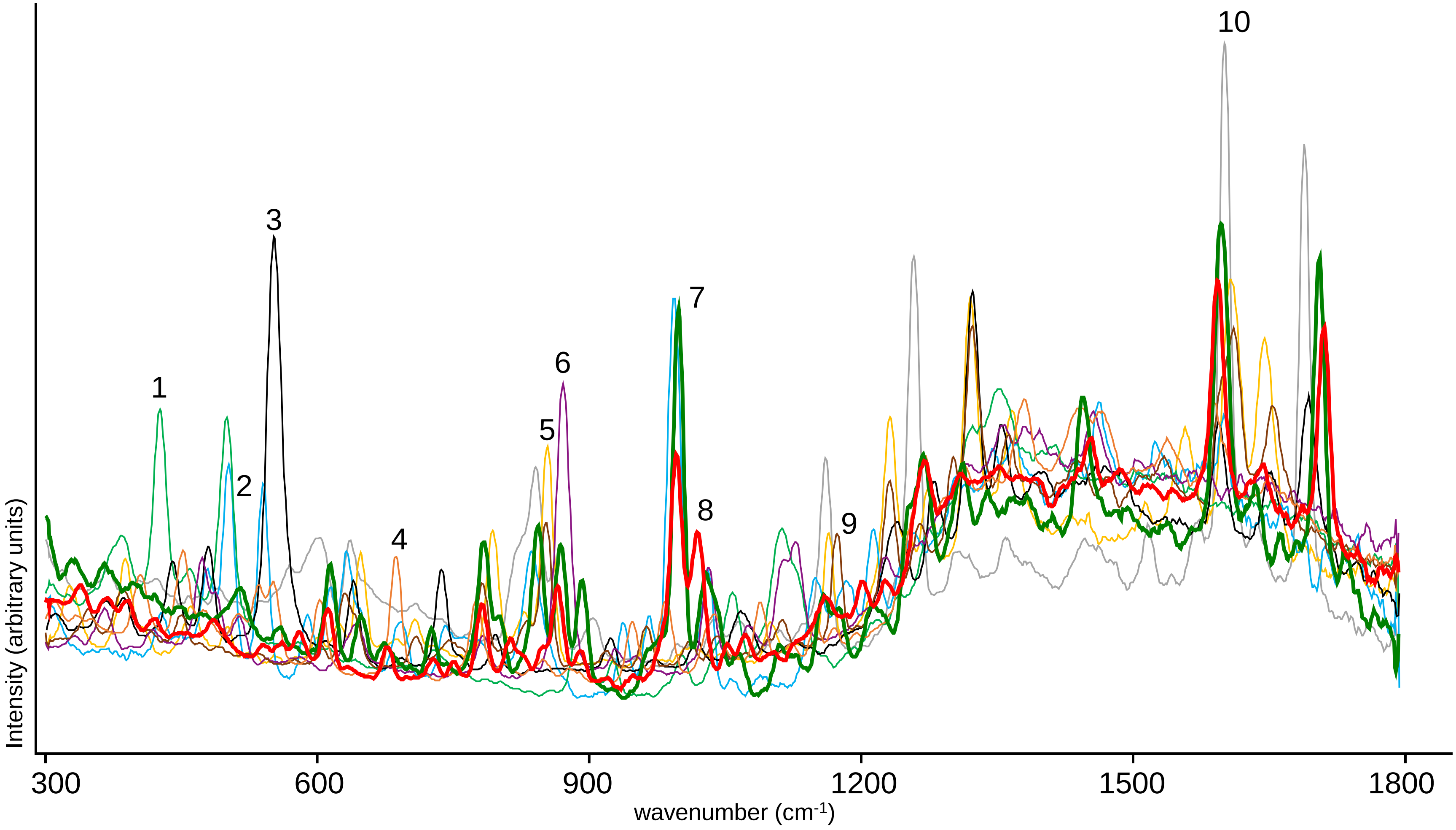

Figure S3. Raman spectra of cocaine and common cutting agents. Inositol (1, light green); levamisole (2, blue); caffeine (3, black); lidocaine (4, orange); paracetamol (5, yellow); mannitol (6, purple), cocaine base (7, green); cocaine HCl (8, red); phenacetine (9, brown); procaine (10, grey). All spectra were pre-processed by SNV and 9 datapoint SG smoothing.
